# Autophagosomes Defeat Ferroptosis by Decreasing Generation and Increasing Discharge of Free Fe^2+^ in Skin Repair Cells to Accelerate Diabetic Wound Healing

**DOI:** 10.1002/advs.202300414

**Published:** 2023-06-30

**Authors:** Shengnan Cui, Xi Liu, Yong Liu, Wenzhi Hu, Kui Ma, Qilin Huang, Ziqiang Chu, Lige Tian, Sheng Meng, Jianlong Su, Wenhua Zhang, Haihong Li, Xiaobing Fu, Cuiping Zhang

**Affiliations:** ^1^ Department of Dermatology China Academy of Chinese Medical Science Xiyuan Hospital Beijing 100091 China; ^2^ Research Center for Tissue Repair and Regeneration Affiliated to the Medical Innovation Research Division The 4th Medical Center of Chinese PLA General Hospital Beijing 100048 China; ^3^ Department of Dermatology Shaanxi Provincial Hospital of Chinese Medicine Xi'an 710003 China; ^4^ Department of the 4th Medical Center of Chinese PLA General Hospital Tianjin Medical University No. 22, Qixiangtai Road, Heping District Tianjin 300070 China; ^5^ Department of the 1th Medical Center of Chinese PLA General Hospital Chinese PLA Medical School 28 Fuxing Road, Haidian District Beijing 100853 China; ^6^ Department of Wound Repair Institute of Wound Repair and Regeneration Medicine Southern University of Science and Technology Hospital Southern University of Science and Technology School of Medicine Shenzhen 518055 China; ^7^ Research Unit of Trauma Care Tissue Repair and Regeneration Chinese Academy of Medical Sciences 2019RU051, 51 Fucheng Road, Haidian District Beijing 100048 China; ^8^ Beijing Key Research Laboratory of Skin Injury Repair and Regeneration 51 Fucheng Road, Haidian District Beijing 100048 China

**Keywords:** autophagosomes, diabetic wounds, endoplasmic reticulum stress, exosomes, ferroptosis

## Abstract

Ferroptosis plays an essential role in the development of diabetes and its complications, suggesting potential therapeutic strategies targeting ferroptosis. Secretory autophagosomes (SAPs) carrying cytoplasmic cargoes have been recognized as novel nano‐warrior to defeat diseases. Here, it is hypothesized that SAPs derived from human umbilical vein endothelial cells (HUVECs) can restore the function of skin repair cells by inhibiting ferroptosis to promote diabetic wound healing. High glucose (HG)‐caused ferroptosis in human dermal fibroblasts (HDFs) is observed in vitro, which results in impaired cellular function. SAPs successfully inhibit ferroptosis in HG‐HDFs, thereby improving their proliferation and migration. Further research show that the inhibitory effect of SAPs on ferroptosis resulted from a decrease in endoplasmic reticulum (ER) stress‐regulated generation of free ferrous ions (Fe^2+^) in HG‐HDFs and an increase in exosome release to discharge free Fe^2+^ from HG‐HDFs. Additionally, SAPs promote the proliferation, migration, and tube formation of HG‐HUVECs. Then the SAPs are loaded into gelatin‐methacryloyl (GelMA) hydrogels to fabricate functional wound dressings. The results demonstrate the therapeutic effect of Gel‐SAPs on diabetic wounds by restoring the normal behavior of skin repair cells. These findings suggest a promising SAP‐based strategy for the treatment of ferroptosis‐associated diseases.

## Graphical abstract

The study demonstrates that SAPs can restore skin repair cell function and promote diabetic wound healing by reducing ER stress‐induced free Fe^2+^ production in HG‐HDFs and stimulating exosome release. A wound dressing containing SAPs loaded in GelMA is developed to enhance the healing process. The results suggest that SAPs have potential for treating diseases associated with ferroptosis.

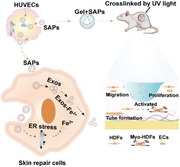

## 1 Introduction

According to the statistics released by the International Diabetes Federation, the number of diabetes patients worldwide is ≈500 million, and this number is expected to reach 755 million by 2045.^[^ ^1^ ^]^ Diabetic wounds are one of the most serious complications of diabetes, from which 19–34% of patients^[^ ^2^ ^]^ greatly suffer, and are a serious economic burden on society.^[^ ^3^ ^]^ A high glucose (HG) environment is a common trigger for diabetic wounds which impairs the function of skin repair cells and delays wound healing.^[^ ^1^, ^4^ ^]^ Despite recent advances in the comprehension of impaired wound healing in diabetes, the pathological and molecular mechanisms remain unclear, and the therapeutic effects of the existing approaches are unsatisfactory.^[^ ^4^ ^]^

Ferroptosis is a newly discovered form of regulatory cell death characterized by an overload of intracellular free ferrous ions (Fe^2+^) and the accumulation of iron‐dependent lipid peroxide. Glutathione peroxidase 4 (GPX4), an antioxidant enzyme negatively regulated by endoplasmic reticulum (ER) stress, removes excess lipid peroxides and is a key upstream regulator of ferroptosis.^[^ ^5^ ^]^ However, in HG environments, the content and activity of the intracellular GPX4 protein are markedly downregulated, leading to excess accumulation of reactive oxygen species (ROS) and Fe^2+^, which are considered critical factors for triggering ferroptosis.^[^ ^6^ ^]^ Ferritin heavy chain 1 (FTH1) is an iron storage protein that regulates intracellular free Fe^2+^ concentrations and reduces Fe^2+^ toxicity resulting from the generation of oxygen radicals by reversibly storing free Fe^2+^.^[^ ^7^ ^]^ However, a previous study found that the expression of FTH1 protein in renal tubule cells (HK‐2 cells) was significantly downregulated in the HG environment.^[^ ^8^ ^]^ Recent studies have shown that skin repair cells, such as human dermal fibroblasts (HDFs) and vascular endothelial cells cultured in HG environments, display reduced cellular functions accompanied by excessive ROS, lipid peroxidation products, and abnormal expression of ferroptosis‐related proteins.^[^ ^9^ ^]^ In a diabetic rat model, direct application of the iron chelator deferoxamine or the ferroptosis inhibitor ferrostatin‐1 on the wound accelerated its healing.^[^ ^10^ ^]^ These reports suggest that ferroptosis is involved in the pathogenesis of diabetic wounds and is a potential therapeutic target for diabetic wound treatment.

Autophagy is a generic term for autophagosome formation and the fusion of autophagosomes with lysosomes.^[^ ^11^ ^]^ Autophagy can modify the angiogenic and oxidative stress abilities of endothelial cells and the aging of fibroblasts.^[^ ^12^ ^]^ Secretory autophagosomes (SAPs), which are generated by secretory autophagy,^[^ ^13^ ^]^ belong to the extracellular vesicles (EVs) family and have emerged as a new mode of intercellular communication by transferring cargo to target cells. Exposure to stress, specifically starvation, enhances secretory autophagy and decreases degradative autophagy by inducing a loss of lysosomal integrity.^[^ ^14^ ^]^ Recent studies on SAPs have focused on unraveling their role in the occurrence and development of diseases, such as cancers^[^ ^15^ ^]^ and acute respiratory distress syndrome (ARDS)^[^ ^16^ ^]^ suggesting a promising strategy for the diagnosis and mechanistic studies of these diseases. Although the pathological effects of SAPs on some diseases have been described, their therapeutic applications, especially in the treatment of diabetic wounds have not been fully explored.

Herein, we hypothesized that SAPs defeat HG‐induced ferroptosis in skin repair cells to accelerate diabetic wound healing and explored the related molecular mechanisms. SAPs were isolated from human umbilical vein endothelial cells (HUVECs) challenged by starvation. After identification, SAPs were used to treat HG‐induced skin repair cells, including HDFs and endothelial cells (substituted with HUVECs in this study). Our results demonstrate that SAPs inhibit ferroptosis and restore the functions of skin repair cells like HG‐HDFs and HG‐HUVECs in vitro. The relevant mechanisms involve a decrease in ER stress‐regulated free Fe^2+^ generation and the discharge of free Fe^2+^ via exosome (Exos) release. SAPs have been loaded into gelatin‐methacryloyl (GelMA) hydrogels to fabricate functional wound dressings. The results showed a better therapeutic effect of SAPs released from GelMA on diabetic wounds by restoring the normal behavior of skin repair cells. So, this research is a successful attempt to apply SAPs for therapeutic applications.

## 2 Results and Discussion

### 2.1 Isolation and Characterization of SAPs

**Figure 1:**
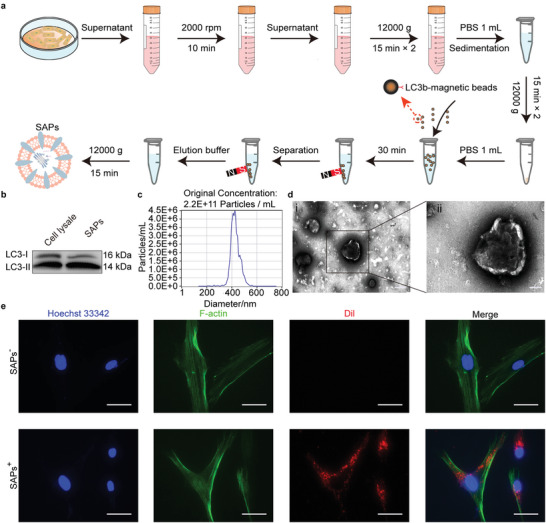
Isolation and characterization of SAPs. a) Schematic diagram showing the isolation process of SAPs. b–d) Characterization of SAPs, including Western blotting analysis(b) for the surface makers, LC3‐I and LC3‐II (*n* = 3), NTA analysis (c) for the size and concentration with ≈2000‐fold dilution (n = 3), and TEM images (d) for the morphology of SAPs (*n* = 3). e) Fluorescent images showing the internalization of SAPs by HG‐HDFs after co‐culturing for 4 h (*n* = 3 per group). Cell nuclei, filamentous actin, and SAPs were labeled with Hoechst‐33342 (blue fluorescence), F‐actin (green fluorescence), and Dil (red fluorescence), respectively. Scale bar, 200 nm for (di), 100 nm for (dii), 25 µm for (e).

SAPs employed in this study were generated via secretory autophagy.^[^ ^17^ ^]^ As shown in Figure S1 (Supporting Information), SAPs with the biomarkers LC3‐I and LC3‐II were generated and secreted from HUVECs by exocytosis under starvation conditions.^[^ ^18^ ^]^ In our study, magnetic beads modified with an LC3b‐antibody (containing LC3‐I and LC3‐II) were employed to capture SAPs from the serum‐free medium of HUVECs after pretreatment with high‐speed centrifugation (**Figure** **1**a). The higher expression ratio of LC3‐II/LC3‐I in isolated SAPs compared to that in cell lysates indicated successful isolation (Figure 1b), which is consistent with previous reports.^[^ ^19^ ^]^ Nanoparticle tracking analysis (NTA) results demonstrated that the separated SAPs displayed a diameter of 415.8 ± 69 nm with a concentration of 2.2 × 10^11^ particles mL^−1^ (Figure 1c), which is consistent with the previous literature.^[^ ^15^ ^]^ Compared with exosomes, the yield of SAPs is much higher, and the acquisition of SAPs does not require sophisticated ultracentrifugation (Figure S2, Supporting Information).^[^ ^15^, ^20^ ^]^ Furthermore, the transmission electron microscopy (TEM) images in Figure 1d and Figure S3 (Supporting Information) show the typical double‐layered membrane morphology of SAPs. The internalization of SAPs is a prerequisite for exerting regulatory functions on target cells. As shown in Figure 1e, 1,1“‐dioctadecyl‐3,3,3”,3'‐tetramethylindocarbocyanine perchlorate‐ (Dil‐) labeled SAPs with red fluorescence distributed uniformly in the cytoplasm (green fluorescence labeled with F‐actin) of HG‐HDFs, indicating that SAPs could be internalized by HG‐HDFs within 4 h. These results suggest that SAPs were successfully isolated from the serum‐free medium of HUVECs and could be internalized by HG‐HDFs.

### 2.2 HG Environments Impaired the Functions of HDFs by Inducing Ferroptosis

Abnormal glucose concentrations in diabetic wounds impair the normal cellular behavior of skin repair cells such as proliferation and migration.^[^ ^6^, ^9^ ^]^ In our study, three glucose concentrations including 5 mM (LG), 25 mM (NG), and 50 mM (HG) were used to investigate the effect of glucose concentration on the cellular behavior of HDFs. We observed that proliferation (Figure , S4a,b Supporting Information) and migration (Figure S4c,d Supporting Information) of HDFs were impaired by treatment with either LG or HG. In particular, the migration area of HG‐treated HDFs was 67.5% less than the NG group when cultured for 24 h. Malnutrition or eutrophication makes HDFs more sensitive to ferroptosis, which hampers their normal cellular functions.^[^ ^21^ ^]^ Therefore, we investigated the ferroptosis‐associated characteristics, including Fe^2+^ and ROS accumulation, in HDFs.^[^ ^22^ ^]^ As shown in Figure S4e,f (Supporting Information), the Fe^2+^ concentration (demonstrated by red fluorescence) in LG‐ and HG‐treated HDFs was much higher than that in NG‐treated HDFs. Similar trends were observed for ROS accumulation (Figure S4g,h Supporting Information). Ferritin heavy chain 1 (FTH1)^[^ ^23^ ^]^ and GPX4)^[^ ^6b^ ^]^ have been reported to be negative regulators of free Fe^2+^ accumulation in cells. In our experiments, the expression of FTH1 and GPX4 was significantly downregulated in LG‐ and HG‐treated HDFs, indicating that the concentration of free Fe^2+^ was significantly increased in the cytoplasm. Collectively, these results demonstrated that adverse glucose environments impair HDF function by inducing ferroptosis.

### 2.3 SAPs Restored Cellular Functions of HG‐HDFs by Inhibiting Their Ferroptosis

**Figure 2:**
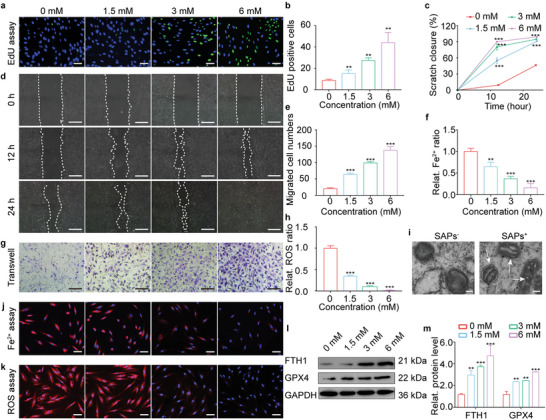
SAPs restored cellular functions of HG‐HDFs by inhibiting their ferroptosis. a,b) EdU immunofluorescence images (a) and corresponding statistical analysis (b) indicating the proliferation behavior of HG‐HDFs treated by SAPs with different concentrations (0, 1.5, 3, and 6 mm) (*n* = 3 per group). Blue and green fluorescence represent Hoechst‐33342 staining and EdU staining, respectively. c) and d) Representative migrating images (d) and quantitative statistics (c) showing scratch closure ratio of HG‐HDFs co‐cultured with SAPs (0, 1.5, 3, and 6 mm) demonstrated by scratch assay. White dotted lines indicated the boundary of migration (*n* = 3 per group). e,g) Representative images (g) and matching statistical analysis (e) showing the migrated cell numbers of HG‐HDFs co‐cultured with SAPs reflected by transwell assay. The migrated HG‐HDFs in the back of the upper chamber of the transwell system were dyed with crystal violet (*n* = 3 per group). f,j) Representative fluorescent images (j) and quantitative analysis (f) of intracellular Fe^2+^ accumulation in HG‐HDFs exposed to SAPs with varying concentrations after 24 h (*n* = 3 per group). Intracellular Fe^2+^ and cell nuclei were dyed with red and blue fluorescence, respectively. h,k) Representative fluorescent images (k) and quantitative analysis (h) showing intracellular ROS level of HG‐HDFs after incubating with SAPs for 24 h (*n* = 3 per group). Intracellular ROS and cell nuclei were dyed with red and blue fluorescence, respectively. i) TEM images showing mitochondrial ultrastructure (represented by white arrows) in HG‐HDFs with or without the treatment of SAPs. l,m) Representative Western blotting images (l) and quantitative band intensities (m) detecting protein expression level of FTH1 and GPX4 in HG‐HDFs stimulated with SAPs after 24 h (*n* = 3 per group, all proteins levels are normalized to loading control, GAPDH). Data represent mean ± SD. Statistical significance was calculated by one‐way ANOVA with Tukey's significant difference multiple comparisons for (b), (c), (e), (f), (h), and (m). Significant differences between 0 mM group with other groups are indicated as **p < 0.01, ***p < 0.001. Scale bar, 50 µm for (a), (j) and (k), 100 µm for (d) and (g).

Interestingly, the introduction of SAPs reversed the effects of HG on the fate of HG‐HDFs. First, we investigated the effect of SAPs on the viability of HG‐HDFs using a CCK‐8 assay (Figure S5, Supporting Information). The viability of HG‐HDFs at all SAPs concentrations was higher than that in the 0 mM group. At first, there was a gradual increase in viability when the SAP concentration increased from 0 to 6 mM followed by a slight decrease at 12 mm. Hence, in subsequent experiments, 6 mm was selected as the maximum SAP concentration. The proliferation and migration abilities of SAPs‐treated HG‐HDFs were analyzed. As shown in **Figure** **2**a,b, the proliferative activity of HG‐HDFs treated with SAPs (6 mm) was enhanced by approximately fivefold compared to that of the control group (0 mm), as detected by the EdU assay. Additionally, the migration area in the scratch assay (Figure 2c,d) and the number of crystal violet‐positive HG‐HDFs in the transwell assay (Figure 2e,g) increased in a dose‐dependent manner, demonstrating that the migration ability of HG‐HDFs was restored by the addition of SAPs. These results indicate that SAPs could restore the proliferation and migration functions of HDFs that were impaired by HG environments. After that, the effect of SAPs on ferroptosis in HG‐HDFs was studied from four perspectives: intracellular Fe^2+^ accumulation, ROS levels, mitochondrial deformation, and the expression of ferroptosis‐related proteins. As shown in Figure 2f,j, a strong red fluorescent signal indicated that large amounts of free Fe^2+^ accumulated in the cytoplasm of HG‐HDFs. However, with increasing SAPs concentration, the red fluorescent signal gradually became faint, and the accumulation of free Fe^2+^ decreased by ≈80% after treatment with 6 mm SAPs. Similar results were also observed for the intracellular ROS levels, where ROS production was markedly reduced and achieved ≈92% attenuation after treatment with 6 mm SAPs (Figure 2h,k). Next, we observed mitochondrial deformation inside HG‐HDFs, as indicated by white arrows in Figure 2i. The vanished mitochondrial cristae caused by the HG environment (labeled SAPs^−^) were recovered after co‐incubation with SAPs (labeled SAPs^+^). We observed that the expression of the ferroptosis‐related proteins FTH1 and GPX4 was upregulated when SAPs concentration increased from 0 to 6 mm, achieving ≈4.3‐ and 3‐fold increases, respectively (Figure 2l,m). The upregulation of FTH1 and GPX4 inside HG‐HDFs indicated that the binding ability to free Fe^2+^ and the efficacy of ROS scavenging in HG‐HDFs was gradually recovered. Collectively, SAPs inhibited ferroptosis in HG‐HDFs, recovering their proliferation and migration activities hampered by the HG environment.

### 2.4 SAPs Inhibited Ferroptosis by Decreasing ER Stress‐Regulated Accumulation of Free Fe^2+^ in HG‐HDFs

**Figure 3:**
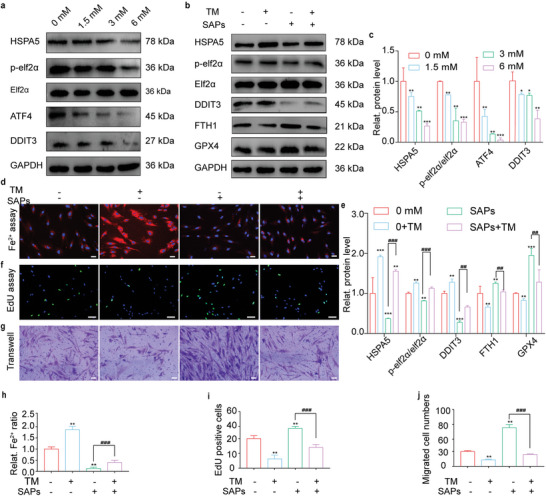
SAPs inhibited ferroptosis by decreasing ER stress‐regulated generation of free Fe^2+^ in HG‐HDFs. a,c) Representative Western blotting images (a) and quantitative band intensities (c) indicating the expression level of ER stress‐associated proteins (HSPA5, p‐elf2*α*/elf2*α*, ATF4, and DDIT3) in HG‐HDFs co‐incubated with SAPs (0, 1.5, 3 and 6 mm) (*n* = 3 per group, all proteins levels are normalized to loading control, GAPDH). b) and e) Representative Western blotting images (b) and quantitative band intensities (e) showing the expression of ER stress‐related proteins (HSPA5, p‐elf2*α*/elf2*α*, DDIT3), and ferroptosis‐associated proteins (FTH1 and GPX4) in HG‐HDFs with or without the treatments of SAPs and TM (the activator of ER stress) (*n* = 3 per group, all proteins levels are normalized to loading control, GAPDH). d,h) Representative fluorescent images (d) and quantitative analysis (h) of intracellular Fe^2+^ level in HG‐HDFs treated with or without SAPs and TM (*n* = 3 per group). f,i) Representative EdU immunofluorescence images (f) and corresponding quantitative analysis (i) of HG‐HDFs treated with or without SAPs and TM (*n* = 3 per group). g,j) Representative images (g) and statistical analysis (j) showing the migrated cell numbers of HG‐HDFs with or without the existence of SAPs and TM reflected by transwell assay (*n* = 3 per group). Results are representative of at least three independent experiments. Data represent mean ± SD. Statistical significance was calculated by one‐way ANOVA with Tukey's significant difference multiple comparisons for (c), (e), (h), (i), and (j). Significant differences between TM^−^+SAPs^−^ group with other groups are indicated as * p < 0.05, **p < 0.01, ***p < 0.001. Significant differences between TM^−^SAPs^+^ group and other groups are indicated as ^##^ *p* < 0.01, *p* < 0.001. Scale bar, 50 µm for (d) and (f), 100 µm for (g).

Excess accumulation of Fe^2+^ in the cytoplasm is the primary cause of ferroptosis,^[^ ^9^ ^]^ which could be avoided by decreasing the generation of free Fe^2+^. As ER stress affects the production of free Fe^2+^,^[^ ^24^ ^]^ we explored whether SAPs could mitigate ER stress in HG‐HDFs and, eventually, decrease the generation of free Fe^2+^. We explored the effects of SAPs on the expression of ER stress‐related proteins. As shown in **Figure** **3**a,c, the expression of ER stress‐related proteins, including the heat shock protein family A member 5 (HSPA5), phospho‐eukaryotic translation initiation factor 2 subunit alpha (p‐elf2*α*), eukaryotic translation initiation factor 2 subunit alpha (elf2*α*), activating transcription factor 4 (ATF4), and DNA‐damage‐inducible transcript 3 (DDIT3), was significantly reduced with increasing concentrations of SAPs. When the concentration of SAPs was 6 mm, the expression levels of HSPA5, ATF4, DDIT3, and the ratio of p‐elf2*α*/elf2*α* were reduced by ≈3.3‐, 9‐, 2‐, and 2‐fold, respectively. These results indicated that ER stress inside HG‐HDFs was mitigated by SAPs in a dose‐dependent manner.

To further explore the role of SAPs in mitigating ER stress‐induced ferroptosis, tunicamycin (TM, an ER stress activator) was added to the HG‐HDF culture medium. The concentrations of TM were chosen based on both ER stress response and cell viability stability. As shown in Figure S6a (Supporting Information), when the concentration of TM was 24 µm, the mRNA expression of DDIT3 and ATF4 was activated. TM with a concentration of 48 µm reduced the cell viability of HG‐HDFs, showing obvious cytotoxicity (Figure S6b, Supporting Information). Hence, 24 µm was chosen as the maximum concentration of TM for subsequent experiments. As shown in Figure 3b,e, the introduction of TM aggravated ER stress in HG‐HDFs by upregulating the expression of ER stress‐associated proteins and decreasing the expression of FTH1 and GPX4 (labeled TM^+^+SAPs^−^). The introduction of SAPs (labeled as TM^−^+SAPs^+^) downregulated the expression of ER stress‐associated proteins and upregulated the expression of FTH1 and GPX4 compared to those in the group of TM^−^+SAPs^−^. However, ferroptosis mitigation by SAPs was partially inhibited when ER stress was augmented by TM (labeled TM^+^+SAPs^+^). SAPs downregulated the expression of ER stress‐associated proteins and upregulated the expression levels of FTH1 and GPX4 to a lesser extent; these functions were restricted in the presence of TM. These results verify that SAPs inhibit ferroptosis in HG‐HDFs by mitigating ER stress.

We observed the effect of SAPs on the accumulation of free Fe^2+^ (Figure 3d,h) inside HG‐HDFs in the presence of TM. Similarly, the introduction of TM (labeled TM^+^+SAPs^−^) significantly upregulated the intracellular Fe^2+^ concentration compared to that in HG‐HDFs (labeled TM^−^+SAPs^−^). Moreover, treatment with SAPs downregulated the accumulation of free Fe^2+^ in TM‐preprocessed HG‐HDFs. Additionally, we investigated the effects of SAPs on the cellular functions of TM‐preprocessed HG‐HDFs. As shown in Figure 3f,g,i,j the proliferative and migratory functions of HG‐HDFs were recovered in the TM^−^+SAPs^+^ group compared to those in the TM^−^+SAPs^−^ group. For HG‐HDFs pretreated with TM, the introduction of SAPs did not recover cell proliferation and migration abilities in the SAPs^+^ group (labeled as TM^−^+SAPs^+^). These results indicate that SAPs can inhibit the production of excess free Fe^2+^ inside HG‐HDFs by mitigating ER stress and restoring the normal cellular functions of HG‐HDFs.

### 2.5 SAPs Inhibited Ferroptosis by Accelerating Exos’ Release to Discharging Free Fe^2+^ from HG‐HDFs

**Figure 4:**
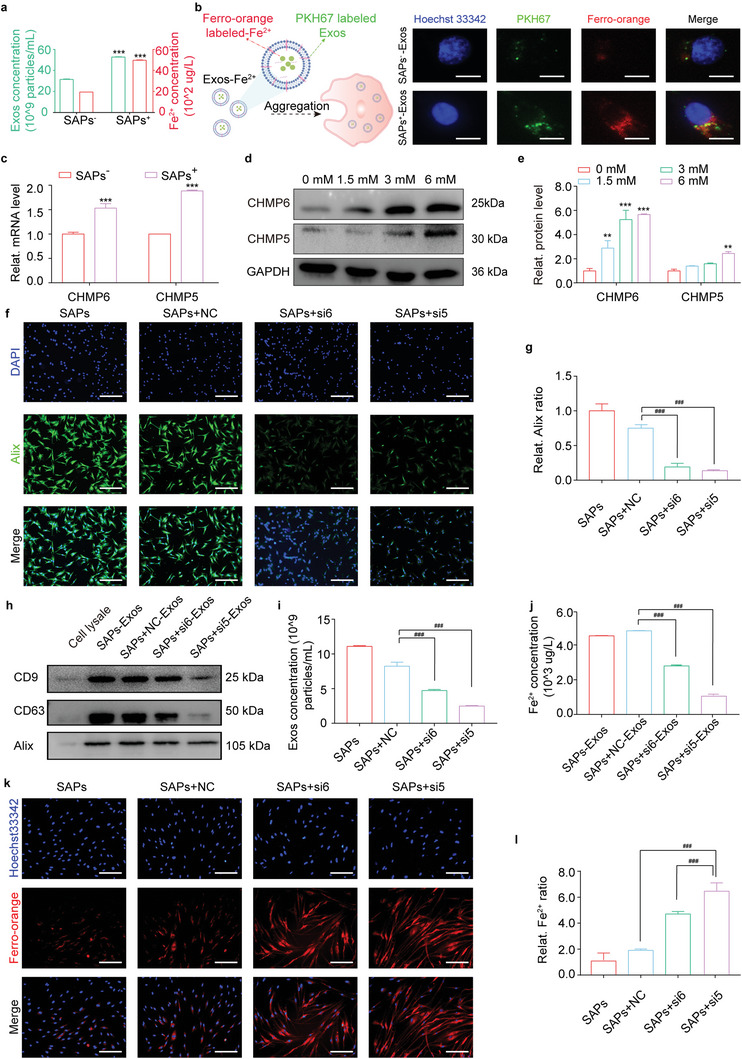
SAPs inhibited ferroptosis by accelerating Exos’ release for discharging free Fe^2+^ from HG‐HDFs. a) Quantitative statistics demonstrating the concentrations of Exos and Fe^2+^ in the Exos derived from HG‐HDFs stimulated with or without SAPs (*n* = 3 per group). b) Schematic diagram and fluorescent images indicating the internalization of Exos containing Fe^2+^ (derived from HG‐HDFs stimulated with or without SAPs) by HUVECs for 2 h. Exos were co‐stained with green PKH67 and red ferro‐orange (*n* = 3 per group). c) qRT‐PCR analysis indicating the gene expression level of CHMP6 and CHMP5 in HG‐HDFs with or without SAPs (*n* = 3 per group, all mRNA levels are normalized to loading control, GAPDH). d,e) Western blotting analysis (d) and corresponding quantitative band intensities (e) displaying the protein expression level of CHMP6 and CHMP5 in HG‐HDFs cultured with SAPs (*n* = 3 per group, all proteins levels are normalized to loading control, GAPDH). f,g) Corresponding fluorescent images (f) and quantitative analysis (g) showing Alix positive HG‐HDFs treated with different groups (SAPs, SAPs+NC, SAPs+si6, SAPs+si5) (*n* = 3 per group). h) Representative Western blotting images showing the expression of biomarkers (CD9, CD63, Alix) on Exos secreted by HG‐HDFs treated with different groups (*n* = 3 per group). i) Quantitative statistics of the concentration of Exos derived from HG‐HDFs treated with different groups demonstrated by NTA analysis (*n* = 3 per group). j) Quantitative analysis of Fe^2+^ concentration inside Exos of HG‐HDFs treated with different groups measured by ICP‐MS (*n* = 3 per group). k,l) Representing fluorescent images (k) and quantitative analysis (l) indicating intracellular Fe^2+^ concentration in HG‐HDFs treated with different groups (*n* = 3 per group). Data represent mean ± SD. Statistical significance was calculated by Students *t*‐test with Welchs correction for (a) and (c), or one‐way ANOVA with Tukey's significant difference multiple comparisons for (e), (g), (i), (j), and (l). Significant differences between SAPs^−^ or 0 mm groups with other groups are indicated as **p < 0.01, *** *p* < 0.001. Significant differences between SAPs+NC group and other groups are indicated as *p* < 0.001. Scale bar,50 µm for (b), 100 µm for (f) and (k).

In addition to reducing the generation of free Fe^2+^, discharging excess Fe^2+^ may be another strategy for inhibiting ferroptosis in HG‐HDFs. Cells eliminate waste by secreting Exos.^[^ ^25^ ^]^ Therefore, we aimed to explore whether SAPs could boost the secretion of Exos, thereby discharging free Fe^2+^ to inhibit ferroptosis in HG‐HDFs. As shown in **Figure** **4**a and Figure S7 (Supporting Information), the concentration of Exos secreted by SAPs‐treated HG‐HDFs was approximately twice that of Exos secreted by untreated HG‐HDFs. Additionally, a higher concentration of Fe^2+^ was observed in Exos derived from SAPs‐treated HG‐HDFs (labeled SAPs^+^), as demonstrated by inductively coupled plasma–mass spectrometry (ICP‐MS) results. To visualize the transportation of free Fe^2+^, Exos were labeled with PKH67 (green fluorescence for Exos) and ferro‐orange (red fluorescence for Fe^2+^) and cocultured with HUVECs for 2 h. As shown in Figure 4b, the green fluorescence of Exos and the red fluorescence of Fe^2+^ were co‐localized in the cytoplasm of HUVECs, indicating that the secreted Exos could carry free Fe^2+^. Moreover, a stronger fluorescence intensity in the colocalization area inside HUVECs was observed for Exos derived from SAPs‐treated HG‐HDFs, demonstrating that treatment with SAPs promoted the secretion of Exos to discharge more Fe^2+^. Furthermore, we used an Exos secretion inhibitor (GW4869) to demonstrate the effect of Exos secretion on the discharging of Fe^2+^. As shown in Figure S8 (Supporting Information), SAPs significantly downregulated the accumulation of free Fe^2+^ inside HG‐HDFs. Interestingly, when Exos secretion was pre‐inhibited by GW4869, the downregulation of free Fe^2+^ by SAPs was moderately restricted (SAPs^+^+GW4869). Collectively, these results indicated that the secretion of Exos could transport free Fe^2+^ from the intracellular to the extracellular domain and that SAPs enhanced this function to discharge free Fe^2+^ from HG‐HDFs.

To explore the underlying mechanisms, we analyzed the intersection of genes enriched in the derivatives of HUVECs (from the Vesiclepedia database) and ferroptosis suppressor genes (from the FerrDb database), as shown in the Venn diagram in Figure S9 (Supporting Information). Among the 29 common targets in the intersection area, two membrane proteins, charged multivesicular body protein 6 (CHMP6) and charged multivesicular body protein 5 (CHMP5), caught our attention because they are recognized as regulators that control the secretion of Exos.^[^ ^26^ ^]^ They are important members of the endosomal sorting complex required for transport III (ESCRT‐III), which promotes the shedding of multivesicular bodies (MVBs) from the cytoplasm and the formation of Exos.^[^ ^27^ ^]^ In our experiment, we observed that the expression levels of CHMP6 and CHMP5 in SAPs‐treated HG‐HDFs were upregulated by ≈1.6‐ and 2‐fold, respectively (Figure 4c). Thereafter, we investigated the effect of SAPs on the expression levels of CHMP6 and CHMP5 (Figure 4d,e). The expression of these two proteins was gradually upregulated following SAPs treatment in a dose‐dependent manner. To verify the role of CHMP6 and CHMP5, four small interfering RNAs (siRNAs), named si6‐1, si6‐2, si5‐1, and si5‐2, were designed to silence the expression of CHMP6 and CHMP5. As shown in Figure S10a–f (Supporting Information), si6‐1 and si5‐1 displayed the highest silencing efficiencies of CHMP6 and CHMP5, respectively, in terms of gene and protein expression. Hence, in subsequent experiments, si6‐1 (labeled si6) and si5‐1 (labeled si5) were chosen to silence the expression of CHMP6 and CHMP5, respectively. Next, we observed the biogenesis of Exos inside HG‐HDFs using immunofluorescence staining for apoptosis‐linked gene‐2 interacting protein X (Alix), which is a marker of MVBs. As shown in Figure 4f,g, the strong green fluorescence of Alix appeared in the SAPs^+^ and SAPs^+^ NC‐treated HG‐HDFs groups, indicating that large amounts of HG‐HDFs produced Exos. With the silencing of CHMP6 and CHMP5 by si6 and si5, respectively, the biogenesis of Exos from HG‐HDFs was heavily restrained, as reflected by the faint green fluorescence. Secreted Exos from the four groups displayed typical biomarkers, including CD9, CD63, and Alix (Figure 4h). With the silencing of CHMP6 and CHMP5, the concentration of secreted Exos decreased by 46.7–71% (Figure 4i; Figure S10g, Supporting Information) compared to that of HG‐HDFs treated with SAPs+NC. We then measured the concentration of free Fe^2+^ inside the Exos from the above groups using ICP‐MS. As shown in Figure 4j, the concentration of Fe^2+^ in Exos from SAPs+si6 and SAPs+si5 treated HG‐HDFs was much lower than that in Exos from SAPs^+^ and SAPs^+^ NC‐treated HG‐HDFs, indicating that inhibiting the secretion of Exos by si6 or si5 impaired the transportation of free Fe^2+^ inside HG‐HDFs. These results indicate that SAPs promote the secretion of Exos that discharge free Fe^2+^ by upregulating the expression of CHMP6 and CHMP5.

To further validate that SAPs relieve ferroptosis in HG‐HDFs through CHMP6 and CHMP5 upregulation, we observed intracellular Fe^2+^ accumulation in SAPs treated HG‐HDFs with or without CHMP5 and CHMP6 silencing. The intracellular Fe^2+^ staining in Figure 4k,l exhibited an opposite trend to the content of Fe^2+^ inside Exos (Figure 4j), indicating that only a small amount of free Fe^2+^ was discharged by Exos after the silencing of CHMP6 and CHMP5 in SAPs‐treated HG‐HDFs. It has been reported that upregulation of CHMP6 and CHMP5 expression increases cell tolerance to ferroptosis.^[^ ^28^ ^]^ Therefore, we examined the viability of HG‐HDFs by silencing CHMP6 and CHMP5 in the presence of SAPs and erastin (a ferroptosis activator). As shown in Figure S11a,b (Supporting Information), SAPs‐treated HG‐HDFs antagonized the ferroptosis‐promoting effects of erastin and displayed high cell viability. However, after pretreatment with erastin and silencing of CHMP6 and CHMP5 by si6‐1 and si5‐1, respectively, almost all HG‐HDFs died after 9 h, further indicating that CHMP6 and CHMP5 play crucial roles in inhibiting ferroptosis in HG‐HDFs. We also examined the migratory ability of HG‐HDFs by silencing CHMP6 and CHMP5 in the presence of SAPs. The results of the scratch and transwell assays indicated that the silencing of CHMP6 and CHMP5 impaired the migration activity of HG‐HDFs, and the scratch area of SAPs+si6 and si5 treated HG‐HDFs was much smaller than that of HG‐HDFs treated with SAPs or SAPs+NC (Figure S11c–f, Supporting Information). These results indicate that the ability of SAPs to recover the cellular behavior of HG‐HDFs was restrained by the silencing of CHMP6 and CHMP5. Collectively, SAPs can inhibit ferroptosis in HG‐HDFs by inducing CHMP6 and CHMP5 mediated‐Exos release to discharge free Fe^2+^.

### 2.6 SAPs Restore Cellular Functions of HG‐HUVECs by Inhibiting Ferroptosis

The formation of granulation tissue composed of neovascularized cells and fibroblasts indicates the transition of the healing process from the inflammatory phase to the proliferative phase.^[^ ^29^ ^]^ However, the HG environment in diabetic wounds impairs the proliferation, migration, and tube‐formation abilities of HUVECs, thereby inhibiting angiogenesis around the wounds.^[^ ^30^ ^]^ Based on the finding that SAPs improved the cellular behaviors of HG‐HDFs, we investigated whether SAPs could restore the cellular functions of HG‐HUVECs. First, we examined the internalization of SAPs by HG‐HUVECs. ​Similar to HG‐HDFs, SAPs were internalized by HG‐HUVECs within 4 h (Figure S12a, Supporting Information). The proliferation ability of HG‐HUVECs treated with SAPs (labeled as SAPs^+^) was upregulated by 50% compared to that of the control group (labeled as SAPs^−^), as shown in Figure S12b,f (Supporting Information). Similar trends were observed in the migration ability of HG‐HUVECs, as demonstrated by the scratch (Figure S12c,g, Supporting Information) and transwell assays (Figure S12d,h, Supporting Information). The scratch area and the number of migrating SAPs‐treated HG‐HUVECs increased approximately twofold and fourfold compared to the control group (labeled as SAPs^−^), respectively. In addition to their proliferation and migration abilities, the effect of SAPs on the tube formation of HG‐HUVECs was also explored. As shown in Figure S12e,k (Supporting Information), the branch points in the SAPs group (labeled as SAPs^+^) increased by approximately ninefold compared to those in the control group (labeled as SAPs^−^). Moreover, we examined ferroptosis‐associated characteristics, including free Fe^2+^ accumulation (Figure S12l,i, Supporting Information), ROS production (Figure S12m,j, Supporting Information), and the expression of FTH1 and GPX4 (Figure S12n,o, Supporting Information). The results showed that SAPs decreased the accumulation of free Fe^2+^ and ROS production, and upregulated the expression of FTH1 and GPX4. These findings suggest that SAPs can restore the cellular behavior of skin repair cells, demonstrating great therapeutic potential during the proliferative phase of diabetic wounds.

### 2.7 Characterization of the Functional Wound Dressing Composed of Porous GelMA Hydrogel Containing SAPs

**Figure 5:**
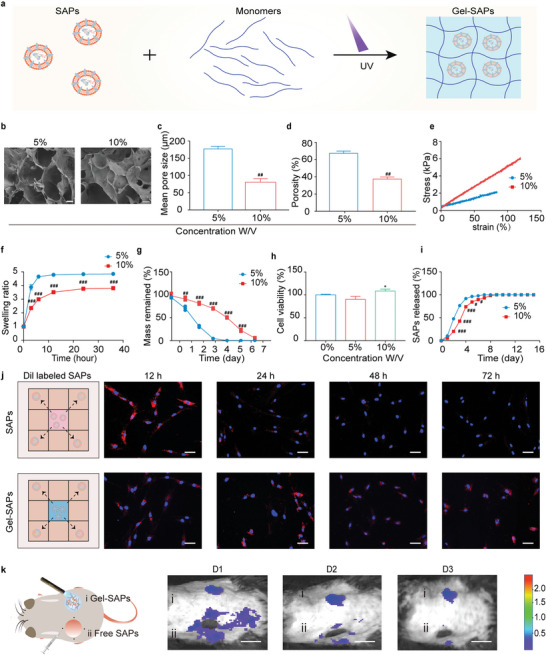
Characterization of a functional wound dressing composed of porous GelMA hydrogel containing SAPs. a) Illustration displaying the prepared process of Gel‐SAPs. b) SEM images showing the porous structure of 5%‐Gel and 10%‐Gel (*n* = 3 per group). c,d) Quantitative statistics of pore size (c) and porosity (d) from SEM images in (b) (*n* = 3 per group). e,f) Stress‐strain curve (e) and swelling behavior (f) of 5%‐Gel and 10%‐Gel (*n* = 3 per group). g) Degradation ratio of 5%‐Gel and 10%‐Gel in vitro by immersing them in 1 mL DPBS containing 0.02 U ML^−1^ collagenase type II (*n* = 3 per group). h) Biocompatibility of 0%, 5% and 10%‐Gel reflected by CCK‐8 assay (*n* = 5 per groups). i) Released curve of SAPs from 5%‐Gel and 10%‐Gel (*n* = 3 per group). j) Illustration and fluorescent images of HG‐HDFs co‐cultured with free SAPs or Gel‐SAPs. SAPs were labeled red fluorescence by Dil and cell nuclei were labeled blue fluorescence by hoechst‐33342 (*n* = 3 per group). k) Illustration and representative images reflecting retention time of Dil labeled free SAPs (four‐point injection) or Gel‐SAPs in wound area at days 1, 2, and 3 (*n* = 6 per group). Results are representative of at least three independent experiments. Data represent mean ± SD. Statistical significance was calculated by Student's *t*‐test with Welch's correction for (c‐g, and i), or one‐way ANOVA with Tukey's significant difference multiple comparisons for (h). Significant differences between 0% groups and other group are indicated as *p < 0.05. Significant differences between 5%‐Gel group and other groups are indicated as ^#^ *p* < 0.05, ^##^ *p* < 0.01, ^###^ *p* < 0.001. Scale bar, 10 µm for (b), 50 µm for (j), 100 µm for (k).

The promising results showing that SAPs can restore the functions of skin repair cells by inhibiting ferroptosis encouraged us to explore the potential of SAPs in promoting diabetic wound healing. Similar to other EVs, the rapid systemic elimination of SAPs in vivo limits their clinical applications.^[^ ^31^ ^]^ Therefore, numerous biomaterials such as microneedles,^[^ ^32^ ^]^ tetrahedral DNA nanostructures,^[^ ^33^ ^]^ and hybrid skin patches ^[^ ^34^ ^]^ can be explored to achieve the sustainable release and even distribution of *Centella asiata*,^[^ ^32^ ^]^ resveratrol,^[^ ^33c^ ^]^ and vascular endothelial growth factors.^[^ ^34^ ^]^ GelMA hydrogels have been recognized as attractive tissue‐engineering materials for the release of exosomes and have shown promising applicability in regenerative medicine.^[^ ^35^ ^]^ To make the SAPs more applicable to wound healing, we employed a porous GelMA hydrogel to load the SAPs under UV photopolymerization (**Figure** **5**a). GelMA hydrogels (labeled Gel) with different monomer concentrations (5% and 10%) were studied. As the monomer concentration increases from 5% to 10%, the crosslinking degree of the Gels increases, leading to a more compact network structure (Figure 5b), smaller pore size (Figure 5c), lower porosity (Figure 5d), and higher robustness (Figure 5e). Additionally, increasing the monomer concentration and crosslinking degree reduced the swelling ratio (Figure 5f) and degradation rate (Figure 5g and Figure S13, Supporting Information) of the Gels. The 10% Gel was degraded after 132 h, which took longer than the 60 h required for the 5% Gel. We assessed the in vivo stability of the Gel‐SAPs by implanting them subcutaneously in mice and observing the residues on D3, D7, and D14 using H&E staining. The results showed that the sheet of the Gel‐SAPs (diameter of 0.5 cm and height of 0.3 cm) gradually degraded over time in vivo, and the Gel‐SAPs was completely degraded at D14 in the subcutaneous embedding model (Figure S14, Supporting Information). Regarding the metabolic pathways of GelMA, the literature suggests that it can be further degraded by matrix metalloproteinases (MMPs) and other collagenases at the wound site, resulting in the production of polypeptides that can be excreted through urine.^[^ ^36^ ^]^ We also evaluated the biocompatibility of the 5% Gel and 10% Gel in vitro using a CCK‐8 assay, with HG‐HDFs cultured without Gel as a control. As shown in Figure 5h, the viability of HG‐HDFs cultured on the 10% Gel was slightly higher than that of cells cultured on the 5% Gel. We speculated that the 10% Gel might be more appropriate for promoting the cellular extension of HG‐HDFs (Figure S15, Supporting Information). This result indicates that the 10% Gel is more appropriate for the growth of HG‐HDFs. The monomer concentration also influenced the release behavior of SAPs, with SAPs being completely released at D5 for the 5% Gel and D8 for the 10% Gel (Figure 5i). The longer release time of the 10% Gel may be due to its denser network structure and smaller pore sizes. Therefore, we selected the 10% Gel for subsequent animal experiments.

We then evaluated whether the process of photocrosslinking and the residual initiator affected the ability of the SAPs to proliferate, migrate, and inhibit ferroptosis. As shown in Figure S16 (Supporting Information), there was no significant difference in the effects of the Gel‐SAP immersion solution (Gel‐SAP‐IS) compared with free SAPs in terms of cell proliferation, migration, iron metabolism, and ROS generation. This suggests that photocrosslinking and the residual initiator did not reduce the SAP activity. Next, we evaluated the retention time of SAPs in the free form SAPs and 10% Gel (Gel‐SAPs) in vitro and in vivo. As shown in Figure 5j, the red fluorescence of DiI‐labeled SAPs released from the Gel‐SAPs was detected after 72 h of co‐incubation with HG‐HDFs, whereas the red fluorescence of free SAPs became very weak after co‐culturing for 48 h. This indicated that the encapsulation of SAPs in the Gel improved their retention time. Intravital imaging in small animal models confirmed these results, showing that the fluorescence intensity of SAPs locally injected by the four‐point injection vanished at D3, whereas the fluorescence in the Gel‐SAP group displayed no significant regression (Figure 5k). These findings suggest that the porous Gel can prolong the release time of the SAPs. Additionally, we assessed the biocompatibility and immunogenicity of the Gel‐SAPs. As shown in Figure S17 (Supporting Information), there was no significant difference in the number of live cells and cytoskeletons of HG‐HDFs and HG‐HUVECs treated with the Gel‐SAPs–IS compared to the NS groups (Figure S17a,b, Supporting Information). Furthermore, there was no obvious difference in the indexes of ALT (Alanine aminotransferase), AST (Aspartate aminotransferase), UREA, CREA (creatinine), CK‐MB (creatine kinase isotype MB), IL‐6 (interleukin‐6), TNF‐*α* (tumor necrosis factor‐*α*), and IFN‐*γ* (interferon‐*γ*) (Figure S17c‐j). The microscopy images of H&E‐stained organ slices showed no obvious histological abnormalities or inflammatory cell infiltration compared to those obtained for the control group (Figure S17k, Supporting Information). Thus, the Gel‐SAPs possess good biocompatibility and low immunogenicity. The hemolysis rates of ddH_2_O, NS‐SAPs, 10% Gel‐SAPs, and 5% Gel‐SAPs were 100%, 1.23%, 1.34%, and 1.36%, respectively (Figure S18, Supporting Information). ​ According to the international standard ISO 10993–4, the hemolysis results revealed that the Gel‐SAPs did not cause hemolysis.^[^ ^37^ ^]^ Collectively, we encapsulated SAPs in a porous Gel and achieved the sustained release of SAPs at the wound site, creating a functional wound dressing called Gel‐SAPs.

### 2.8 Gel‐SAPs Promoted Diabetic Wound Healing In Vivo by Inhibiting Ferroptosis of Skin Repair Cells

**Figure 6:**
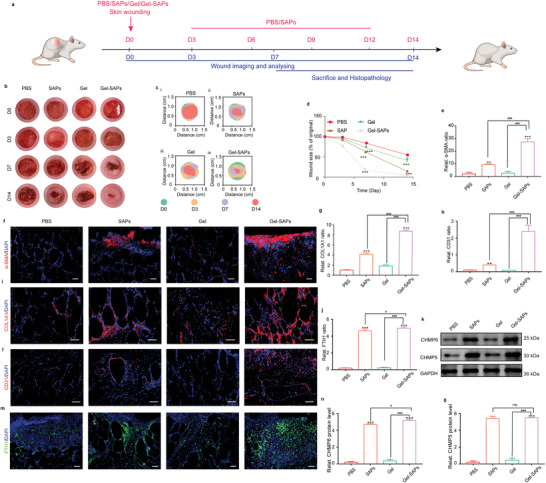
Gel‐SAPs promoted diabetic wound healing in vivo by inhibiting the ferroptosis of skin repair cells. a) Timeline of wound treatment process. b–d) Representative images (b) closure traces (c) and quantitative analysis (d) of wound closure at days 0, 3, 7, and 14 treated with PBS, SAPs, Gel, and Gel‐SAPs (*n* = 6 per group). e,f) Immunofluorescence staining images (f) and quantification of *α*‐SMA (e) (red fluorescence) at wound sites with the treatment of PBS, SAPs, Gel, and Gel‐SAPs for day 7 (*n* = 6 per group). g,i) Immunofluorescence staining images (i) and quantification of COL1A1 (g) (red fluorescence) at wound sites with the treatment of PBS, SAPs, Gel, and Gel‐SAPs for day 7 (*n* = 6 per group). h,l) Immunofluorescence staining images (l) and quantification of CD31 (h) (red fluorescence) at wound sites with the treatment of PBS, SAPs, Gel, and Gel‐SAPs for day 7 (*n* = 6 per group). j,m) Representative fluorescent images (m) and quantitative analysis (j) of FTH1 level in PBS, SAPs, Gel, and Gel‐SAPs groups for day 7. (*n* = 6 per group). k,n,o) Representative Western blotting images (k) and quantitative band intensities (n,o) indicating the expression level of CHMP6 and CHMP5 in PBS, SAPs, Gel, and Gel‐SAPs groups. (*n* = 6 per group, all proteins levels are normalized to loading control, GAPDH). The cell nuclei were dyed blue fluorescence with 4',6‐diamidino‐2‐phenylindole (DAPI) for (f), (i), (l), and (m). Results are representative of at least three independent experiments. Data represent mean ± SD. Statistical significance was calculated by one‐way ANOVA with Tukey's significant difference multiple comparisons for (d), (e), (g), (h), (j), (n), and (o). Significant differences between PBS groups and other group are indicated as ***p < 0.001. Significant differences between Gel‐SAPs group and other groups are indicated as ^#^ *p* < 0.05, *p* < 0.001 compared with other groups. NS, not significant. Scale bar, 100 µm for (f) and (m), 50 µm for (i) and (l).

First, we investigated the promoting effects of SAPs (37.5, 75, and 150 mm)‐encapsulated GelMAs on wound healing. As shown in Figure S19 (Supporting Information), the healing rates were 73.28%, 97.68%, and 89.31% at D10 for the 37.5, 75, and 150 mm treated groups, respectively. Therefore, 75 mm was selected as the optimal concentration for subsequent animal experiments. Considering that the complete metabolism time of free SAPs is D3 in vivo as shown in Figure 5k, the animals were administered 15 mm SAPs at 2‐day intervals. To evaluate the ability of Gel‐SAPs to accelerate wound healing in DB/db mice, a round full‐skin defect wound (with a diameter of ≈1 cm) was created on the backs of the diabetic mice, with PBS, free SAPs, and Gel used as the control groups (**Figure** **6**a). As shown in Figure 6b–d and Figure S20 (Supporting Information), wounds treated with Gel‐SAPs contracted markedly compared to those treated with PBS, free SAPs, or Gel at D3 after injury. At D14, the area of the wounds treated with Gel‐SAPs decreased by ≈87%, whereas only ≈65%, 84%, and 70% reductions were observed in the groups treated with PBS, free SAPs, and Gel, respectively. Furthermore, we utilized a panoramic view of H&E to assess the wound size in PBS, free SAPs, Gel, and Gel‐SAPs on D7 and D14 (Figure S21a(i),c(i), Supporting Information). The results showed that the size of the wound decreased to 0.27 cm on D7, and the wound was almost invisible on D14 in the Gel‐SAPs‐treated group. These findings are consistent with the results of the optimal images, wound closure trace, and quantitative analysis shown in Figure 6b–d.   Additionally, high‐magnification views of the wound edges were obtained using H&E and Masson's staining of the wound tissues on D7 (Figure S21a,S21aii,b, Supporting Information) and D14 (Figure S21cii,d, Supporting Information) to evaluate the formation of granulation tissue and collagen deposition. Histological analysis revealed that Gel‐SAPs significantly reduced the wound size and increased the thickness of granulation tissue and collagen deposition compared with the other groups.

Additionally, the *α*‐SMA‐mediated contraction of myo‐HDFs is crucial for wound healing.^[^ ^38^ ^]^ We observed that Gel‐SAPs upregulated α‐SMA expression on day D7 after injury. As shown in Figure 6e,f, the red fluorescence signal of SMA was the strongest in Gel‐SAPs‐treated wounds, indicating that many HDFs were activated to myo‐HDFs at D7, and the healing process entered the proliferative phase. Furthermore, the strong red fluorescence signal in Figure 6g,i indicated that activated myo‐HDFs began to secrete large amounts of COL1A1, forming a collagen network at D7. In contrast, a faint red fluorescence signal was observed in PBS‐, free SAPs‐, or Gel treated wounds, indicating insufficient COL1A1 deposition. These results are consistent with those shown in Figure  S21b,d (Supporting Information). Angiogenesis at the wound site was analyzed using anti‐CD31 staining.^[^ ^39^ ^]^ The red fluorescence of CD31 in the Gel‐SAPs was stronger in the wound area than in the control groups, indicating that the slow release of SAPs promoted neovascularization in diabetic wounds (Figure 6h,l).

Finally, the wound tissues collected from the four groups were stained with FTH1 to evaluate the level of ferroptosis in diabetic wounds. As shown in Figure 6j,m, the intensity ratio of green fluorescence in the SAPs and Gel‐SAPs groups was higher than that in the control group (PBS and Gel). We also examined the protein expression of CHMP6 and CHMP5 in the PBS, SAPs, Gel, and Gel‐SAPs groups. The introduction of SAPs significantly increased the expression of CHMP6 and CHMP5 (Figure 6k,n,o). These results indicated that treatment with Gel‐SAPs and SAPs could inhibit the ferroptosis of skin repair cells in vivo, whereas treatment with PBS and Gel could not mitigate the ferroptosis of cells. Therefore, these in vivo results indicated that the sustained release of SAPs from Gel‐SAPs could promote diabetic wound healing by inhibiting ferroptosis and restoring the normal behavior of skin repair cells in the DB/db mouse model.

## 3 Conclusion

**Figure 7:**
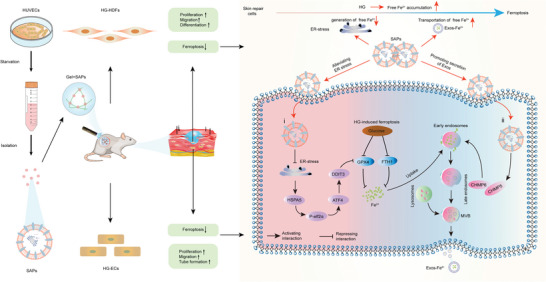
Underlying mechanisms of SAPs to inhibiting ferroptosis of HG‐impaired skin repair cells. i) SAPs downregulated the production of free Fe^2+^ by reducing ER stress. ii) SAPs promoted the secretion of Exos to discharge Fe^2+^ by upregulating the expression of CHMP5/CHMP6.

In this study, we report that SAPs can restore cellular behavior by inhibiting ferroptosis of skin repair cells in diabetic wounds. Our results demonstrated the cascade strategy involved in this process (**Figure** **7**). SAPs can limit the generation of Fe^2+^ by mitigating ER stress or promote the discharge of Fe^2+^ by triggering Exos secretion. To prepare SAPs suitable for wound healing, we encapsulated SAPs in a porous GelMA hydrogel to create functional wound dressings (Gel‐SAPs). Gel‐SAPs can accelerate wound healing by rescuing cellular ferroptosis, promoting the activation of HG‐HDFs, and supporting angiogenesis, collagen deposition, and re‐epithelialization. This study demonstrates that SAPs provide an alternative approach to developing next‐generation therapies for the treatment of ferroptosis‐associated diseases.

## 4 Experimental Section

### Materials

Opti‐modified Eagle's medium (Opti‐MEM) and lipofectamine 2000 were purchased from Invitrogen (USA). Fetal bovine serum (FBS) and penicillin‐streptomycin solution (PS) were obtained from Gibco (USA). PBS, Dulbecco'smodified Eagle medium (DMEM), ethylene diamine tetraacetic acid (EDTA) solution, hematoxylin & eosin (H&E), Masson's trichome staining, cell counting kit‐8 (CCK‐8), radio immunoprecipitation assay lysis buffer (RIPA lysates), bicinchoninic acid protein assay kit (BCA), and Hoechst 33 342 were bought from Solarbio (China). Calcein‐am/propidium iodide (Calcein‐AM/PI) kit, 5‐ethynyl‐2‐deoxyuridine (EdU) kit, 1,1″‐dioctadecyl‐3,3,3″,3″‐tetramethylindocarbocyanine perchlorate (Dil), and polyvinylidene fluoride (PVDF) membrane were obtained from Beyotime (China). Heat shock protein family A member 5 (HSPA5), DNA‐damage inducible transcript 3 (DDIT3), and ferritin heavy chain1(FTH1) were obtained from Cell Signaling Technology (CST, USA). Glutathione peroxidase 4 (GPX4), CD63, CD9, collagen type I alpha 1 chain (COL1A1), *α*‐smooth muscle actin (*α*‐SMA), apoptosis‐linked gene‐2 interacting protein X (Alix), and charged multivesicular body protein 6 (CHMP6) were bought from Abclonal (China). Phospho‐eukaryotic translation initiation factor 2 subunit alpha (p‐elf2*α*), eukaryotic translation initiation factor 2 subunit alpha (elf2*α*), and activating transcription factor 4 (ATF4) were purchased from Proteintech (USA). 4″,6‐Diamidino‐2‐phenylindole (DAPI), CD31, and charged multivesicular body protein 5 (CHMP5) were purchased from Abcam (UK). Porous methacrylate gelatin (GelMA) was gotten from Engineering from life (EFL, China). RNA isolation kit was obtained from YI SHAN (China). One‐step reverse transcription‐PCR (one‐step RT‐PCR) kit was purchased from TAKARA (Japan). Ferro‐orange was purchased from DojinDo (Japan). Magnetic beads were form Absin (China). Erastin and tunicamycin (TM) were purchased from Selleck (USA). The matrigel was from BD Biosciences (USA). Triton X‐100 was purchased from Sigma (Germany). The small interfering RNAs (siRNAs) of CHMP5 and CHMP6 were purchased from Tsingke Biotechnology (China). All reagents were taken for direct use without further treatment.

### Cell Culture

The cell lines of human dermal fibroblasts (HDFs, CRL‐4053, USA) and human umbilical vein endothelial cells (HUVECs, CRL‐1730, USA) were purchased from the American Type Culture Collection (ATCC). HDFs were cultured in DMEM, containing 10% FBS, 1% PS, and different final concentrations of glucose (5 mm for LG, 25 mm for NG, 50 mm for HG) for 60 days. HUVECs were cultured in DMEM containing 10% FBS, 1% PS, and 50 mm glucose. HUVECs were cultured in DMEM containing 1% PS to collect SAPs. The second cells were used for the following experiments.

### Isolation, Purification, and Characterization of SAPs

The isolation and purification of SAPs were according to previous literature.^[^ ^15^ ^]^ Briefly, HUVECs and their cellular debris were removed after centrifugation (2000 rpm, 10 min). The supernatants were collected and centrifuged twice (12 000 × *g*, 15 min) to harvest crude products containing SAPs. Then, the crude products were resuspended with 1 mL PBS and centrifuged twice (12 000 × *g*, 15 min). The sediment was resuspended with 1 mL PBS again and incubated with LC3b‐labeled magnetic beads (1:100, CST, 3868T) for 30 min. A magnetic rack was employed to isolate magnetic beads which absorbed SAPs. Then, the isolated magnetic beads were washed by elution buffer and the supernatant was centrifuged (12 000 × *g*, 15 min) to obtain purified SAPs. The size and concentration of SAPs were determined and analyzed by nanoparticle tracking analysis (NTA, UK). The morphology of SAPs was observed by transmission electron microscopy (TEM, JEOL, Japan). The surface markers of SAPs were analyzed by Western blotting(see Section S1.15, Supporting information).S1.15

### Cell Internalization Assay

SAPs were stained by Dil according to the manufacturer. HG‐HDFs and HG‐HUVECs were seeded in 24‐well plates. When the cells attached to the walls, 15 mm SAPs were added into the culture medium of HG‐HDFs and HG‐HUVECs co‐incubated for 4 h. Then, the cell nuclei and cytoskeleton were stained by Hoechst 33342 (blue fluorescence) and F‐actin (green fluorescence), respectively.

### Cell Viability

For CCK‐8 assay, HG‐HDFs were seeded in 96‐well plates and co‐cultured with SAPs (0, 1.5, 3, 6, 12 mm) for 24 h or TM (Selleck, S7894) (0, 3, 6, 12, 24, 48 µm) for 6 h, respectively. Subsequently, 10 µL of CCK‐8 kit (Solarbio, CA1210) was added into each well. After incubating for 2 h, the absorbance of 450 nm was measured with microplate reader (SpectraMax M3). For live/dead staining assay, HG‐HDFs were transfected with transfection reagents containing 500 µL Opti‐MEM, 5 µL lipofectamine 2000, and 5 µL si‐6/si‐5/nonsence negative control (NC) for 6 h. Then, 3 mm SAPs were added into transfected HG‐HDFs and co‐cultured for 24 h, followed by adding 10 µm erastin (Selleck, s7242). Live cells, dead cells, and cell nuclei were stained with Calcein‐AM (green fluorescence), PI (red fluorescence), and Hoechst 33342 (blue fluorescence), respectively.

### Cell Proliferation Assay

EdU assay was performed according to the instructions of EdU kit (Beyotime, C0071S). HDFs or HG‐HUVECs were seeded in 24‐well plates and attached to the walls. For the effect of glucose, HDFs were cultured and passaged in DMEM containing different concentrations of glucose (LG, NG, and HG) for 60 days. For the effect of SAPs, HG‐HDFs were cultured with SAPs (0, 1.5, 3, and 6 mm) for 24 h. HG‐HUVECs were co‐cultured with 3 mm SAPs for 24 h. For the effect of ER stress, HG‐HDFs were co‐cultured with SAPs (3 mm, 24 h) and TM (24 µm, 6 h) in an orthogonal design. Then, EdU reagent diluted by DMEM (1:1000, v/v) was added into wells and incubated for 12 h at 37 °C. After that, cells were fixed with paraformaldehyde for 15 min and permeabilized for 10 min, followed by co‐culturing with EdU reaction solution (green fluorescence) for 30 min. The representative pictures were taken with a fluorescence microscope (Nikon Eclipse E800, Japan).

### Cell Migration Assay

For the scratch assay, HDFs or HG‐HUVECs were seeded in 12‐well plates. When the cells attached to the walls and cell confluence was up to 90%, the pipette tip (200 µL) was used to create a scratch. After that, HDFs were treated with different concentrations of glucose; SAPs (3 mm), SAPs+NC, SAPs+si‐6, and SAPs+si‐5; or different concentrations of SAPs; and HG‐HUVECs treated with or without 3 mm SAPs were evaluated. Images were taken under a light microscope at 0, 12, and 24 h after scratch. The migrated area was measured by *Image J* software. For transwell assay, cells including HG‐HDFs treated by SAPs with different concentrations (0, 1.5, 3, and 6 mm) for 24 h; HG‐HDFs co‐cultured with SAPs (3 mm) for 24 h and TM (24 µm) for 6 h in an orthogonal design or HUVECs with or without 3 mM SAPs for 24 h were evaluated. Cells with different treatments were seeded in the upper chamber of transwell with 8.0 µm polycarbonate membrane, while the lower chambers were filled with 500 µL DMEM. After 12 h or 24 h, cells in the upper chamber were removed with a cotton swab and cells on the back of the upper chamber were stained by 0.5% crystal violet for about 30 min. Images were taken under a light microscope (Nikon Eclipse E800, Japan), and the migrated cell numbers were quantified by *Image J* software.

### Tube Formation Assay of HUVECs

Matrigel (BD Biosciences, 356237) was dropped into 96‐well plates (50 µL for each well) and polymerized at 37 °C for 45 min. Then, HUVECs (1 × 10^4^ cells per well) were seeded in the matrigel‐covered wells and treated with 0 or 3 mm SAPs for 2 h. Next, the light microscope was employed to obtain tube formation images. Total branching points were calculated through *Image J* software.

### Determination of Intracellular Free Fe^2+^ and ROS Level

For Fe^2+^ determination, HDFs or HUVECs were seeded in 24‐well plates with the following treatments: different concentrations of glucose; different concentrations of SAPs; SAPs (3 mm) and TM (24 µm) in an orthogonal design; SAPs (3 mm); SAPs+NC, SAPs+si‐6, and SAPs+si‐5; SAPs (3 mm) and GW4869 (24 µm) in an orthogonal design. After the treatments, cells were incubated with 10 µm ferro‐orange (red fluorescence) at 37 °C for 30 min. For ROS determination, HDFs or HUVECs were seeded in 24‐well plates with the following treatments: different concentrations of glucose and different concentrations of SAPs. After the treatments, cells were incubated with 10 µm DHE at 37 °C for 30 min. Cell nuclei were counterstained with Hoechst 33342 dye. The representative images were taken with a fluorescence microscope.

### TEM Analysis of the Mitochondrial Ultrastructure Inside HG‐HDFs

HG‐HDFs treated with or without 3 mm SAPs were fixed with 2.5% glutaraldehyde for 4 h and washed twice with PBS. The cells were dehydrated by gradient dehydration. Samples were embedded in epoxy resin, followed by slicing. The mitochondrial structure was observed by TEM.

### Isolation of Exos and Detection of Fe^2+^ Contents in Exos In Vitro

For the isolation of Exos, equal supernatant volume of HG‐HDFs treated by 0 mm SAPs, 3 mm SAPs, SAP+NC, SAP+si6, and SAP+si5 were collected. The extraction and determination of Exos were operated according to the literature.^[^ ^40^ ^]^ The size and concentration of Exos were determined and analyzed by NTA. Biomarkers (CD9, CD63, and Alix) on the surface of Exos were detected by Western blotting. For detection of Fe^2+^ contents in Exos, Exos from different groups were digested by aqua regia solution for 2 h, and free Fe^2+^ in Exos were measured with an Agilent 7800x ICP‐MS spectrometer (USA). For the visualization of Fe^2+^ in Exos, Exos derived from 0 or 3 mm SAP‐treated HG‐HDFs were co‐stained by PKH67 (green fluorescence) and ferro‐orange (red fluorescence) according to the manufacturer. Next, Exos were co‐incubated with HUVECs for 2 h. The cell nuclei of HUVECs were stained by Hoechst 33342.

### Identification of CHMP6 and CHMP5

The intersection between genes enriched in HUVECs‐derivates from Vesiclepedia database (http://www.microvesicles.org/) and the suppressor genes in ferroptosis from FerrDb database (http://www.zhounan.org/ferrdb) were analyzed using the online tool Venn diagram (http://bioinformatics.psb.ugent.be/webtools/Venn/). These genes may serve as promising candidates for further studies regarding potential in the process of inhibiting ferroptosis by SAPs in HG‐HDFs.

### Transient Transfection

HG‐HDFs were seeded in a six‐well plate. Five microliters of siRNAs including si6‐1, si6‐2, si5‐1, and si5‐2 were added to 250 µL Opti‐MEM and then mixed with 5 µL lipofectamine 2000 (Invitrogen, 11668019) in 250 µL Opti‐MEM (Invitrogen, 31985062) for 20 min at room temperature (RT) to prepare transfection reagent. The transfection reagent was added into HG‐HDFs when HG‐HDFs were attached to the walls and co‐incubated for 48 h. The transfection efficiency was measured and evaluated by quantitative real‐time PCR (qRT‐PCR) and Western blotting. siRNA sequences are listed in Table S1 (Supporting Information). A nonsense siRNA duplex was used as a silencer NC.

### Analysis of qRT‐PCR

RNA was extracted by an RNA isolation kit from YI SHAN Biotechnology (Shanghai, 19331ES08). In brief, the spin columns filled with the cell‐lysis solution were centrifuged at the speed of 12 000 × *g* for 1 min. Then, the spin columns were washed by washing buffer and centrifuged at the speed of 12 000 × *g* for 1 min. Next, the columns were transferred to new EP tubes and diluted by DEPC water, followed by centrifugation (12 000 × *g*, 1 min). A One‐Step RT‐PCR kit from TAKARA (RR055A) was used to perform qRT‐PCR. The sequences of primers are listed in Table S2 (Supporting Information).

### Western Blotting Assay

Briefly, RIPA lysates (Solarbio, R0010) were employed to extract proteins. The protein content was quantified by BCA kit. Then, electrophoresis was performed at 120 V for 90 min. After electrophoresis, proteins were transferred onto PVDF membrane by electroblot and blocked by 5% milk for 1 h. After that, PVDF membranes were co‐incubated with primary antibodies including LC3b (1:1000), GPX4 (1:1000, Abclonal, A1933), FTH1 (1:1000, CST, 4393), HASP5 (1:1000, CST, 3177), p‐elf2*α* (1:1000, Proteintech, 28740‐1‐AP), elf2*α* (1:1000, Proteintech, 11170‐1‐AP), ATF4 (1:1000, Proteintech, 10835‐1‐AP), DDIT3 (1:1000, CST, 2895T), CHMP5 (1:1000, Abcam, ab96273), CHMP6 (1:1000, Abclonal, A4975), Alix (Abclonal, A2215), CD63 (Abclonal, A19023), CD9 (Abclonal, A19027), and GAPDH (1:10 000, Abcam, ab181602). Next, the membranes were washed with PBS and incubated with horse radish peroxidase (HRP)‐conjugated secondary antibodies (1:10 000, ZSGB‐BIO, China) for 1 h at RT. Antibody reactivity was detected by the ECL kit (Solarbio, China) and visualized by the UVITEC Alliance MINI HD9 system (UVITEC, Britain). *Image J* software was used to quantify the gray value representing the protein expression level.

### Structural and Physical Properties of Porous GelMA Hydrogels (Gel) and Gel Containing SAPs

Gel with 5% or 10% monomer concentrations (5%‐Gel and 10%‐Gel) were employed in this study. Gels containing 15 mm SAPs were polymerized with UV‐light (*λ* = 365 nm) for 15 s and swollen at PBS for 24 h at 37 °C. The cross‐section morphologies of Gels after the procedure of freeze‐drying were characterized with SEM (ZEISS Gemini 300, Germany). The pore size of Gels was counted from SEM images by Image J software. The porosity of Gels was calculated as Equation (1):

(1) $$\mathrm{Porosity}=\left[\left(W_{1}-W_{2}\right)/\rho V\right]\times 100\%$$

W_1_: the initial weight of Gel, W_2_: the weight of Gel after being immersed in ethanol for 24 h, *ρ*: the density of ethanol, V: the volume of the *GelMA* hydrogel

For tensile test, 5%‐Gel and 10%‐Gel were prepared and polymerized in a mold (5 cm × 2 cm × 0.3 cm). Tensile tests of the samples were conducted on a universal testing machine (CMT6103, China) with the speed of 1 mm min^−1^. The stress–strain curve was recorded during the test. For swelling rate, the frozen‐dried 5%‐Gel and 10%‐Gel were placed in 3 mL PBS and weighed at 24 h. The swelling rate was calculated according to Equation (2). For degradation of Gels, the pieces of the swollen 5%‐Gel and 10%‐Gel were incubated in DPBS containing type II collagenase (0.02 U mL^−1^) at 37 °C for 0, 12, 24, 36, 60, 84, 108, and 132 h. The degradation rate was calculated according to Equation (3).

(2) $$\mathrm{Swelling}\mathrm{rate}\left(\%\right)\;=\frac{W_{2}-W_{1}}{W_{1}}\times 100\%$$

W_1_: the initial weight of Gel, W_2_: the weight of Gel at a different time.

(3) $$\mathrm{Wx}\left(\%\right)=\;\frac{W_{1}-\left(W_{1}-W_{2}\right)}{W_{1}}\times 100\%$$

W_1_: the initial weight of Gel, W_2_: the weight of Gel at a different time, W_X_: The weight remained.

### Biocompatibility Assay of Gel and Gel‐SAPs In Vitro

The biocompatibility of 5%‐Gel and 10%‐Gel was evaluated by culturing HG‐HDFs (2000 cells per well) on the above Gels. The HG‐HDF cells cultured without hydrogel were defined as the control group. CCK‐8 solution was added at 72 h after incubation and cultured for another 2 h. Then, the absorbance at 450 nm was obtained by SpectraMax M3. To evaluate the elongation of HG‐HDFs on Gels, 5%‐Gel and 10%‐Gel were added into 24‐well plates, followed by seeding HG‐HDFs (1 × 10^5^ cells per well) on them. After culturing for 24 and 72 h, the cell nuclei were stained by DAPI (blue fluorescence) and the cytoskeleton was stained by F‐actin (green fluorescence) that reflected the elongation ability of HG‐HDFs. To access the biocompatibility of SAPs on HG‐HDFs and HG‐HUVECs, calcein‐AM/PI double staining and cytoskeleton staining were performed. The cells were treated with Gel‐SAPs immersion solution (Gel‐SAPs‐IS) or NS for 24 h, and then live/dead reagents were added to the cells for 30 min. The cell morphology was visualized by staining F‐actin with Alexa Fluor 488‐conjugated phalloidin. Fluorescence microscopy was used to examine the cells and photograph them.

### The Release Ratio of Gel‐SAPs In Vitro and Vivo

To investigate the release of SAPs from 5%‐Gel and 10%‐Gel in vitro, 15 mm SAPs were mixed with 1 mL 5%‐Gel or 10%‐Gel and cured for 10 s. Then, Gels containing SAPs were immersed into 1 mL PBS. The released SAPs were quantified by NanoPhotometer (IMPLEN, Germany). Six male BKS‐Dock Leprem2Cd479 (DB/db, 7 weeks old) mice were employed to evaluate the release of free SAPs and Gel‐SAPs in vivo by small animal living imaging system. Full‐thickness skin wounds (diameter of ≈1 cm) were created on both sides of the backbone of the same mouse. The wound on the left side was treated with free SAPs (Dil stained) by four‐point injection and the wound on the right side was smeared and cured with Gel‐SAPs (*n* = 6). The release of SAPs was recorded by the small animal living imaging system at 24, 48, and 72 h.

### The Degradation of Gel‐SAPs In Vivo

Nine male DB/db mice (7‐week‐old) were used to access the degradation of Gel‐SAPs in vivo. The 10% Gel‐SAPs (diameter of 0.5 cm and height of 0.3 cm) were placed on a spatula and inserted into subcutaneous pockets on each side of the back of anesthetized mice. The wound area was covered with an oxygen‐permeable wound dressing (3M) after incision. White light photographs and histological analysis was performed on D3, D7, and D14 after the insertion of the 10% Gel‐SAPs (*n* = 6).

### The Biocompatibility and Immunogenicity of Gel‐SAPs In Vivo

Twelve male DB/db mice (7 weeks old) were used to evaluate the biocompatibility and immunogenicity of Gel‐SAPs in vivo (*n* = 6). Mice were divided into two groups equally and randomly (*n* = 6) and received intraperitoneal injection: NS (100 µL), Gel‐SAPs immersion solution (Gel‐SAPs‐IS, 75 mm SAPs loaded in 100 µL GelMA). After D14, the peripheral blood samples of mice were collected from the mice for biochemical assays (ALT, AST, UREA, CREA, and CK‐MB), Elisa assay (IL‐6, TNF‐*α*, and IFN‐*γ*), and major organs (Liver, Heart, Spleen, Lung, and Kidney) of mice were analyzed by H&E staining.

### Bioactivity Comparison Between Released SAPs and Free SAPs

The Gel‐SAPs were incubated in serum‐free DMEM at 37 °C. After 24 h, the DMEM co‐cultured with Gel‐SAPs, referred as Gel‐SAPs‐IS, was collected. The concentration of released SAPs was quantified using a NanoPhotometer (IMPLEN, Germany). The effects of Gel‐SAPs‐IS on cell proliferation, migration, and the inhibition of ferroptosis in HG‐HDFs were accessed, taking free equivalent SAPs as control.

### Hemolytic Properties of the Gel‐SAPs

The hemolytic properties of Gel‐SAPs were evaluated using whole blood samples collected in Eppendorf tubes containing ddH_2_O, NS‐SAPs, 10% Gel‐SAPs, and 5% Gel‐SAPs. After incubation at room temperature for 2 h, the Eppendorf tubes were photographed and recorded, and then centrifuged at 3000 rpm for 10 min. The amount of hemoglobin released into the supernatant was measured using a microplate spectrophotometer at 540 nm.

### Wounding and Treating of Skin Defect Model in Mice

Thirty‐six male DB/db (7 weeks old) mice and 12 C57/BL6JOlaHsd (C57/BJ6, 7 weeks old) male mice were purchased from the SPF Biotechnology Co. Ltd (Beijing, China). All animal experiments were approved by the Institutional Animal Care and Use Committee of Chinese People's Liberation Army General Hospital and reared under standard experimental animal feeding conditions (reference number:2021KY009–HS003). The mice were employed to conduct experiments after 1 week of adaptive feeding. They were anesthetized with sodium pentobarbital (50 mg kg^−1^) and shaved. The full‐thickness skin wounds (diameter of ≈1 cm) were created on both the left and right sides of their backs using a round hole punch. Twelve C57/BJ6 mice were randomly divided into two groups, A and B, using a random number table (*n* = 6). Group A was treated with 0 mm SAPs (100 µL of 10% Gel, applied to the left wound) and 37.5 mm SAPs (37.5 mm SAPs diluted in 100 µL of 10% Gel, applied to the right wound), while Group B was treated with 75 mm SAPs (75 mm SAPs diluted in 100 µL of 10% Gel, applied to the left wound) and 150 mm SAPs (150 mm SAPs diluted in 100 µL of 10% Gel, applied to the right wound). The wound area of each group was photographed and recorded at D0, D2, D5, and D10. Meanwhile, the 36 DB/db mice were randomly assigned to two groups (*n* = 6), C and D. Group C received PBS (100 µL, applied to the left wound) and SAPs (15 mm SAPs diluted in 100 µL of PBS, applied to the right wound), while Group D received Gel (100 µL of 10% Gel, applied to the left wound) and Gel‐SAPs (75 mm SAPs diluted in 100 µL of 10% Gel, applied to the right wound). PBS and SAPs were injected subcutaneously at four points around the wounds at D0 with a dosing interval of every 3 days, while Gel and Gel‐SAPs were smeared on the wound area at D0. The images of wounds were taken at days 0, 3, 7, and 14.

### Histological Analysis

After the mice were sacrificed, wound tissues were peeled and fixed with 4% paraformaldehyde. Then, wound tissues were dehydrated gradually and embedded in paraffin. The embedded wound tissues were cut into sections with 5 µm thickness and stained with hematoxylin and eosin (H&E) kit (Solarbio, G1120), Masson's kit (Solarbio, G1340).

### Immunofluorescence Staining

For Alix staining, HG‐HDFs were co‐incubated with SAPs (3 mm), SAPs+NC, SAPs+si‐6, and SAPs+si‐5 for 48 h, respectively. Then, HG‐HDFs were fixed with 4% paraformaldehyde and permeabilized with 0.2% triton X‐100 (Sigma, 93443). Next, the surface of HG‐HDFs was blocked with 1% bovine serum albumin (BSA) for 1 h and incubated at 4 °C overnight with the primary antibody and with the secondary antibody at RT for 60 min. For immunofluorescence histochemistry staining of COL1A1, CD31, *α*‐SMA, and FTH1, after the procedures of dewaxing and hydration, the antigen repair of sections was conducted using the microwave thermal and citrate buffer. After that, the sections were incubated with anti‐CD31 (1:100, Abcam, ab281586), COL1A1 (1:100, Abclonal, A16891), *α*‐SMA (1:100, Abclonal, A17910), and FTH1 (1:200, CST, 4393) at 4 °C for 12 h.

### Statistical Analysis

GraphPad Prism 8.4.3 (GraphPad Software) was used to analyze and plot all experimental data. The data are presented as mean ± standard deviation (mean ± SD), and each experiment was performed with at least three biological replicates. The number of independent experiments is indicated in the figure legend. Differences between the two groups were analyzed using Student's *t*‐tes/t. One‐way ANOVA analysis was used to analyze differences among multiplegroups followed by a Fisher's protected least significance difference test. Specific statistical details for each experiment can be found in the corresponding figure legends. *p* < 0.05 were considered to be statistically significant.

## Conflict of Interest

The authors declare no conflict of interest.

## Supporting information

Supporting Information

## Data Availability

The data that support the findings of this study are available from the corresponding author upon reasonable request.
