# Supplementary material for: Autophagosomes Defeat Ferroptosis by Decreasing Generation and Increasing Discharge of Free Fe2+ in Skin Repair Cells to Accelerate Diabetic Wound Healing
Source: Adv Sci (Weinh). 2023 Jun 30;10(25):2300414. doi: 10.1002/advs.202300414 (PMC10477857; doi:10.1002/advs.202300414)
Supplement: Supplementary file 1 — Supporting Information [file ADVS-10-2300414-s001.pdf]

ADVANCED SCIENCE
Open Access

Supporting Information

for *Adv. Sci.*, DOI 10.1002/advs.202300414

Autophagosomes Defeat Ferroptosis by Decreasing Generation and Increasing Discharge of Free Fe^{2+} in Skin Repair Cells to Accelerate Diabetic Wound Healing

Shengnan Cui, Xi Liu, Yong Liu, Wenzhi Hu, Kui Ma, Qilin Huang, Ziqiang Chu, Lige Tian, Sheng Meng, Jianlong Su, Wenhua Zhang, Haihong Li, Xiaobing Fu* and Cuiping Zhang**

Autophagosomes defeat ferroptosis by decreasing generation and increasing discharge of free Fe^{2+} in skin repair cells to accelerate diabetic wound healing

Shengnan Cui[1], Xi Liu[2], Yong Liu[3], Wenzhi Hu[2], Kui Ma[2], Qilin Huang[4], Ziqiang Chu[2,5], Lige Tian[4], Sheng Meng[2], Jianlong Su[2], Wenhua Zhang[2], Haihong Li[6,*], Xiaobing Fu[2,5,7,8,*], Cuiping Zhang[2,5,7,8,*]

[1]Department of Dermatology, China Academy of Chinese Medical Science, Xiyuan Hospital, Beijing, 100091, China.

[2]Research Center for Tissue Repair and Regeneration Affiliated to the Medical Innovation Research Division and the 4th Medical Center of Chinese PLA General Hospital, Beijing, 100048, China.

[3]Department of Dermatology, Shaanxi Provincial Hospital of Chinese Medicine, Xi'an, 710003, China.

[4]Department of the 4th Medical Center of Chinese PLA General Hospital, Tianjin Medical University, No. 22, Qixiangtai Road, Heping District, Tianjin, 300070, China.

[5]Department of the 1th Medical Center of Chinese PLA General Hospital, Chinese PLA Medical School, 28 Fuxing Road, Haidian District, Beijing, 100853, China.

[6]Department of Wound Repair, Institute of Wound Repair and Regeneration Medicine, Southern University of Science and Technology Hospital, Southern University of Science and Technology School of Medicine, Shenzhen, 518055, China.

[7]Research Unit of Trauma Care, Tissue Repair and Regeneration, Chinese Academy of Medical Sciences, 2019RU051, 51 Fucheng Road, Haidian District, Beijing, 100048, China.

[8]Beijing Key Research Laboratory of Skin Injury, Repair and Regeneration, 51 Fucheng Road, Haidian District, Beijing, 100048 China.

***Corresponding author:**
Haihong Li, MD, PhD; E-mail: lihaihong1051@126.com
Xiaobing Fu, MD, PhD; E-mail: fuxiaobing@vip.sina.com
Cuiping Zhang, MD, PhD; E-mail: zcp666666@sohu.com
Keywords: autophagosomes, ferroptosis, endoplasmic reticulum stress, exosomes, diabetic wounds

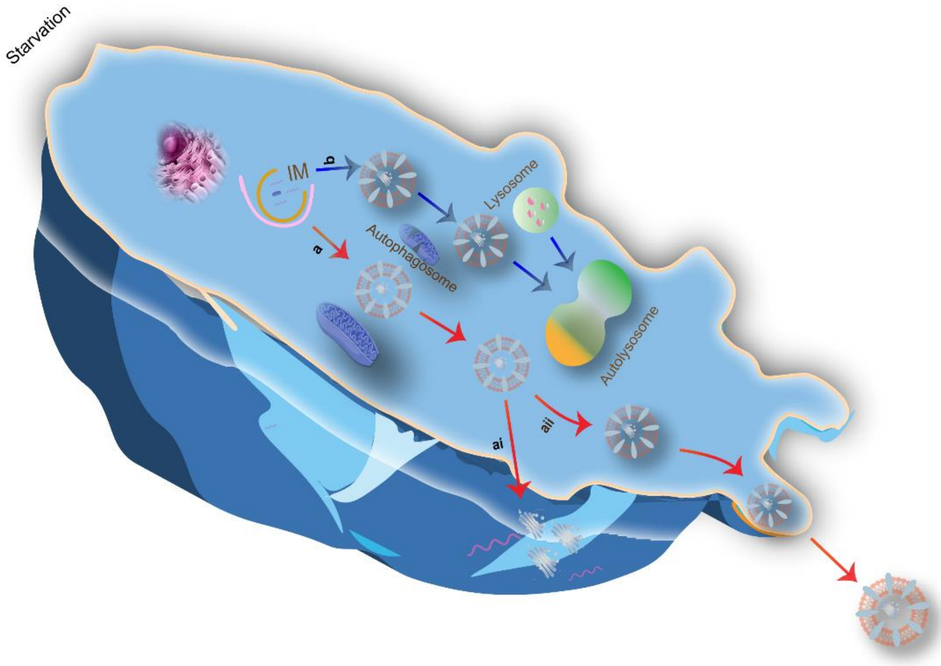

Figure S1. Schematic indicating the formation process of SAPs from HUVECs cultured with serum-free medium. (a) Schematic of secretory autophagy. (ai) The contents of SAPs were released outside the cell membrane, but the membrane of SAPs was left inside the cells. (aii) Intact SAPs were released outside the cells. (b) Schematic representing degradation autophagy.

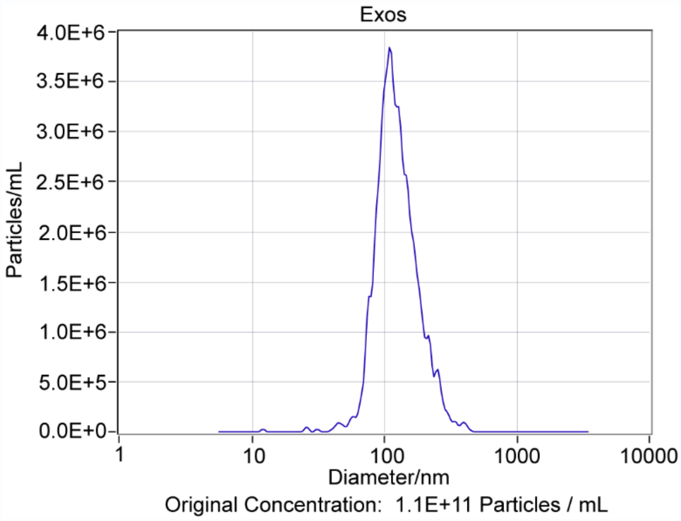

Figure S2. NTA analysis for the size and concentration of Exos (n = 3).

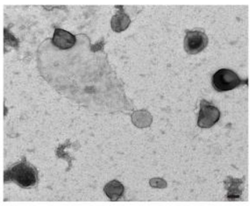

Figure S3. The TEM images for the morphology of SAPs. Scale bar = 200 nm (n = 3).

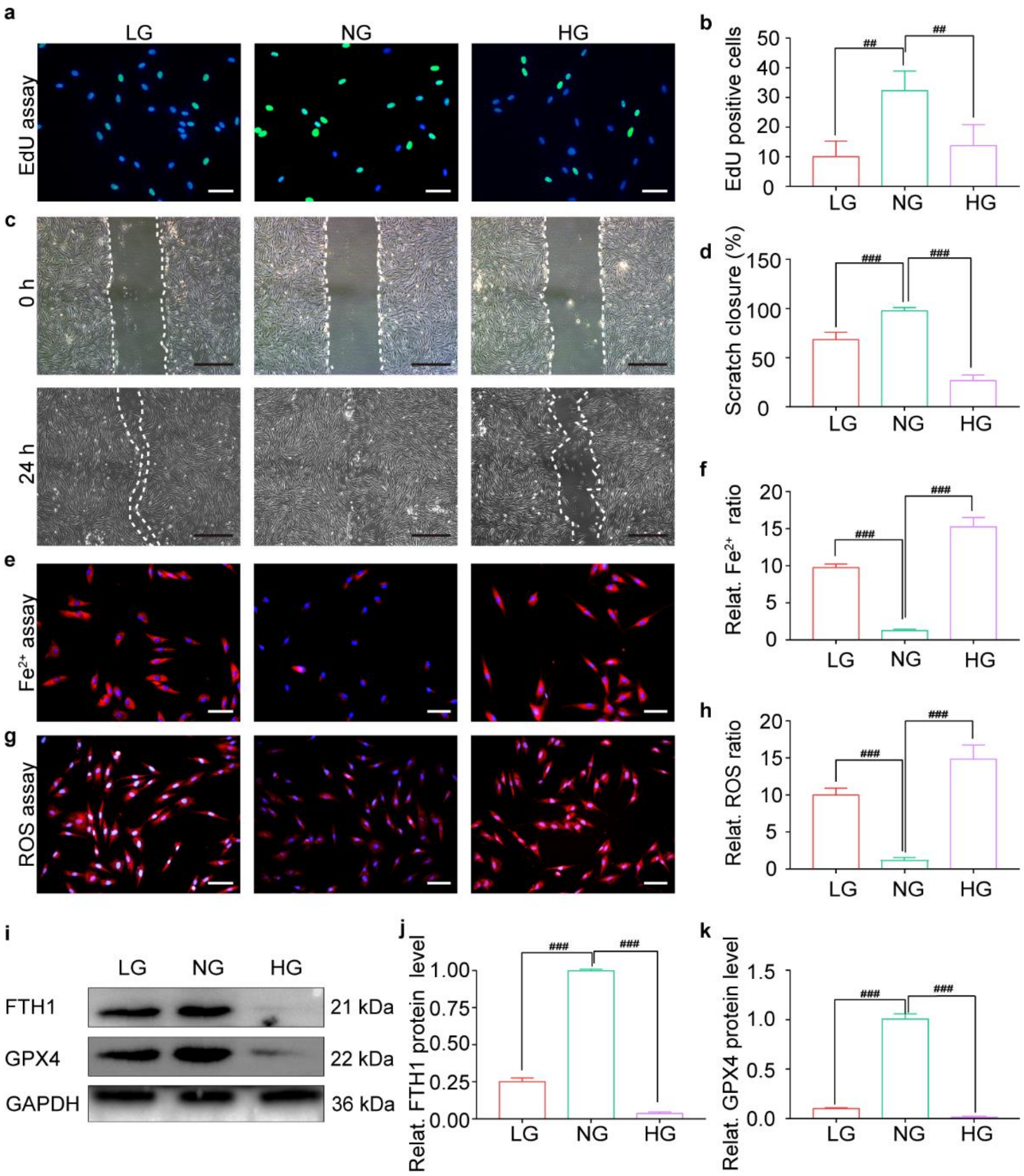

Figure S4. HG impaired cellular functions of HDFs by inducing ferroptosis. a) and b) EdU immunofluorescence images (a) and statistical analysis (b) reflecting proliferation activities of HDFs cultured by cell medium with different concentrations of glucose (LG, NG, and HG) (*n* = 3 per group). c) and d) Representative images (c) and statistical analysis (d) displaying wound closure ratio of HDFs with LG, NG and HG demonstrated by scratch assay. White dotted lines indicated the boundary of migration (*n* = 3 per group). e) and f) Representative fluorescent images demonstrated by ferro-

orange (e) and quantitative analysis (f) of intracellular Fe^{2+} level inside HDFs treated by LG, NG, and HG (n = 3 per group). g) and h) Representative fluorescent images (g) and quantitative analysis (h) of intracellular ROS level in HDFs treated by LG, NG and HG demonstrated by DHE staining (n =3 per group). i) to k) Western blotting analysis (i) and quantitative band intensities displaying the expression of FTH1 (j) and GPX4 (k) in HDFs treated by LG, NG and HG (n = 3 per group, all proteins levels are normalized to loading control, GAPDH). Data represent mean ± SD. Statistical significance was calculated by one-way ANOVA with Tukey's significant difference multiple comparisons for (b), (d), (f), (h), (j) and (k). Significant differences between NG group with other groups are indicated as $^{\#\#}p < 0.01$, $^{\#\#\#}p < 0.001$. Scale bar, 50 μm for (a), (e) and (g), 100 μm for (c).

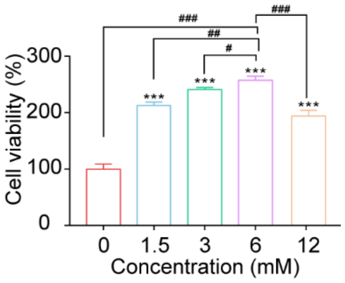

Figure S5. Effects of SAPs on cell viability of HG-HDFs demonstrated in by CCK-8 assay (n = 5 per group). Data represent mean ± SD. Statistical significance was calculated by one-way ANOVA with Tukey's significant difference multiple comparisons. Significant differences between 0 mM groups with other groups are indicated as $^{***}p < 0.001$. Significant differences between 6 mM group and other groups are indicated as $^{\#}p < 0.05$, $^{\#\#}p < 0.01$, $^{\#\#\#}p < 0.001$.

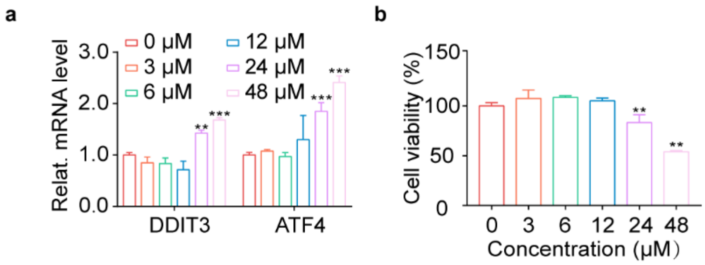

Figure S6. Effects of TM concentrations on ER stress and cell viability of HG-HDFs. a) qRT-PCR analysis indicating the gene expression level of DDIT3 and ATF4 in HG-HDFs treated with different concentrations of TM (*n* = 3 per group, all mRNA levels are normalized to loading control, GAPDH). b) CCK-8 analysis indicating the cell viability in HG-HDFs treated with different concentrations of TM (*n* = 5 per group). Data represent mean ± SD. Statistical significance was calculated by one-way ANOVA with Tukey's significant difference multiple comparisons. Significant differences between 0 mM group with other groups are indicated as $^{**}p < 0.01$, $^{***}p < 0.001$.

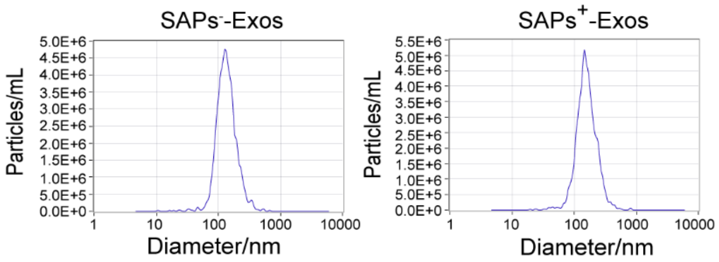

Figure S7. NTA analysis reflecting size distribution and concentration of Exos derived from HG-HDFs with or without SAPs. The present concentrations of Exos from SAPs$^-$ or SAPs$^+$ were diluted by PBS (about 300 folds and 480 folds, respectively) (n = 3 per group).

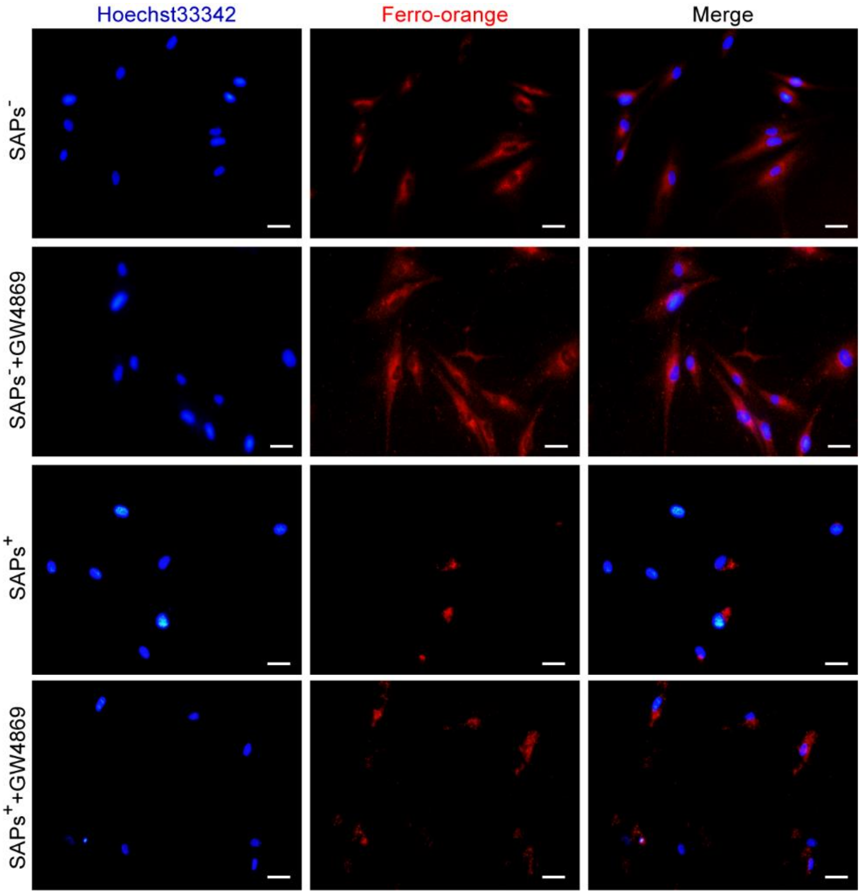

Figure S8. Fluorescent images reflecting intracellular Fe^{2+} accumulation in HG-HDFs with or without SAPs and GW4869 (the inhibitor of Exos' secretion) (*n* = 3 per group). The intracellular Fe^{2+} accumulation was reflected by red fluorescence. scale bar = 50 μm.

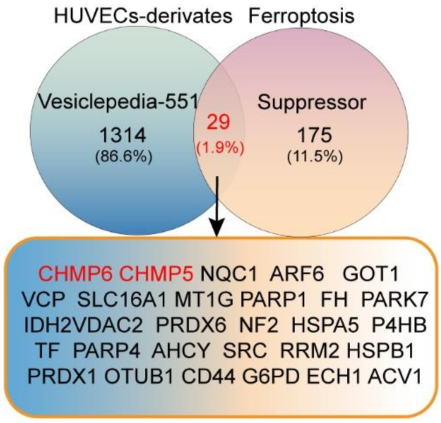

Figure S9. Overlap of mRNA transcripts which enriched in HUVECs-derivates and reported suppressors in ferroptosis analyzed by Venn diagram analysis.

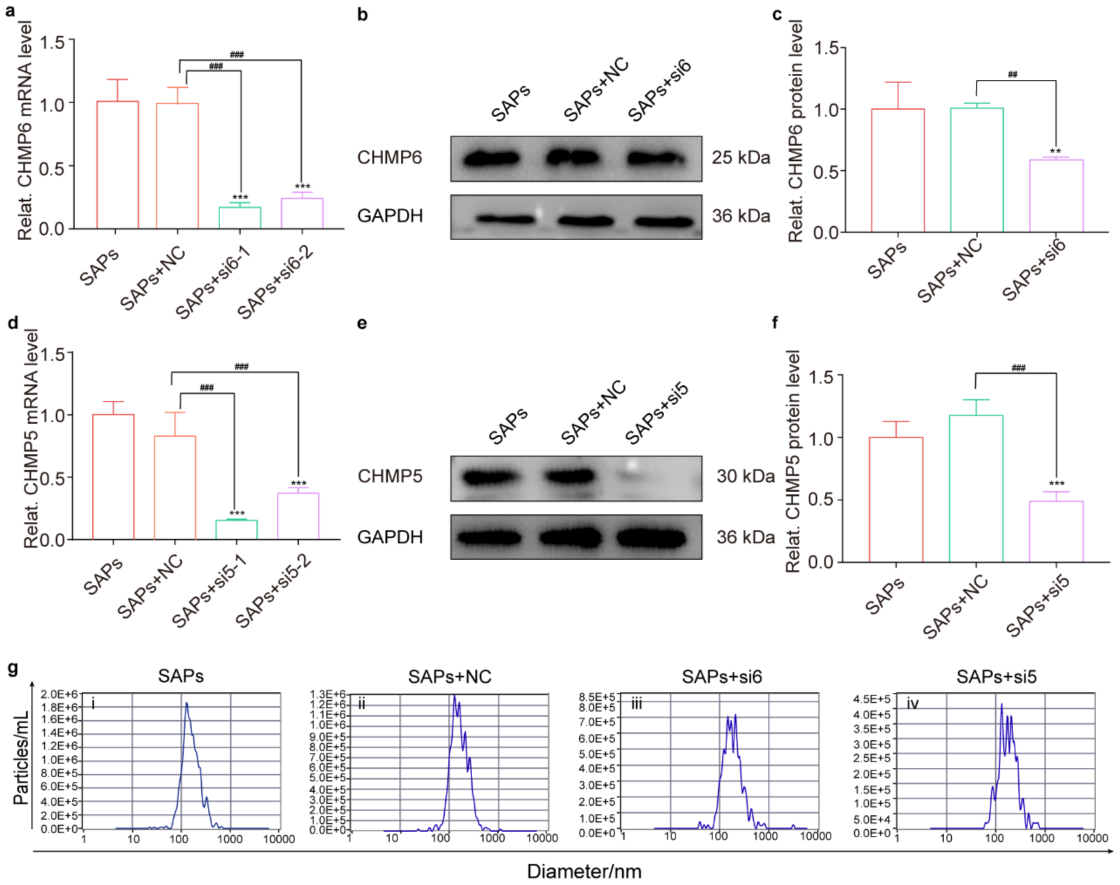

Figure S10. Assessment of the siRNA silencing efficiency and the concentration of Exos derived from HG-HDFs treated by SAPs, SAPs+NC, SAPs+si6, SAPs+si5. a) to c) qRT-PCR analysis (a), representative Western blotting images (b) and quantitative statistics (c) of corresponding band intensities showing the silencing efficiency of CHMP6 in HG-HDFs treated with SAPs ($n = 3$ per group, all mRNA and proteins levels are normalized to loading control, GAPDH). d) to f) qRT-PCR analysis (d), representative Western blotting images (e) and quantitative statistics of corresponding band intensities (f) showing the silencing efficiency of CHMP5 in HG-HDFs treated with SAPs ($n = 3$ per group, all mRNA and proteins levels are normalized to loading control, GAPDH). g) The size distribution and concentration of Exos derived from HG-HDFs treated by SAPs (i), SAPs+NC (ii), SAPs+si6 (iii), SAPs+si5 (iv) The present

concentration data were diluted about 240-fold by PBS in all groups (n = 3 per group). Data represent mean ± SD. Statistical significance was calculated by one-way ANOVA with Tukey's significant difference multiple comparisons for (a), (c), (d) and (f). Significant differences between SAPs group with other groups are indicated as $^{**}p < 0.01$, $^{***}p < 0.001$. Significant differences between SAPs+NC group with other groups are indicated as $^{\#\#}p < 0.01$, $^{\#\#\#}p < 0.001$.

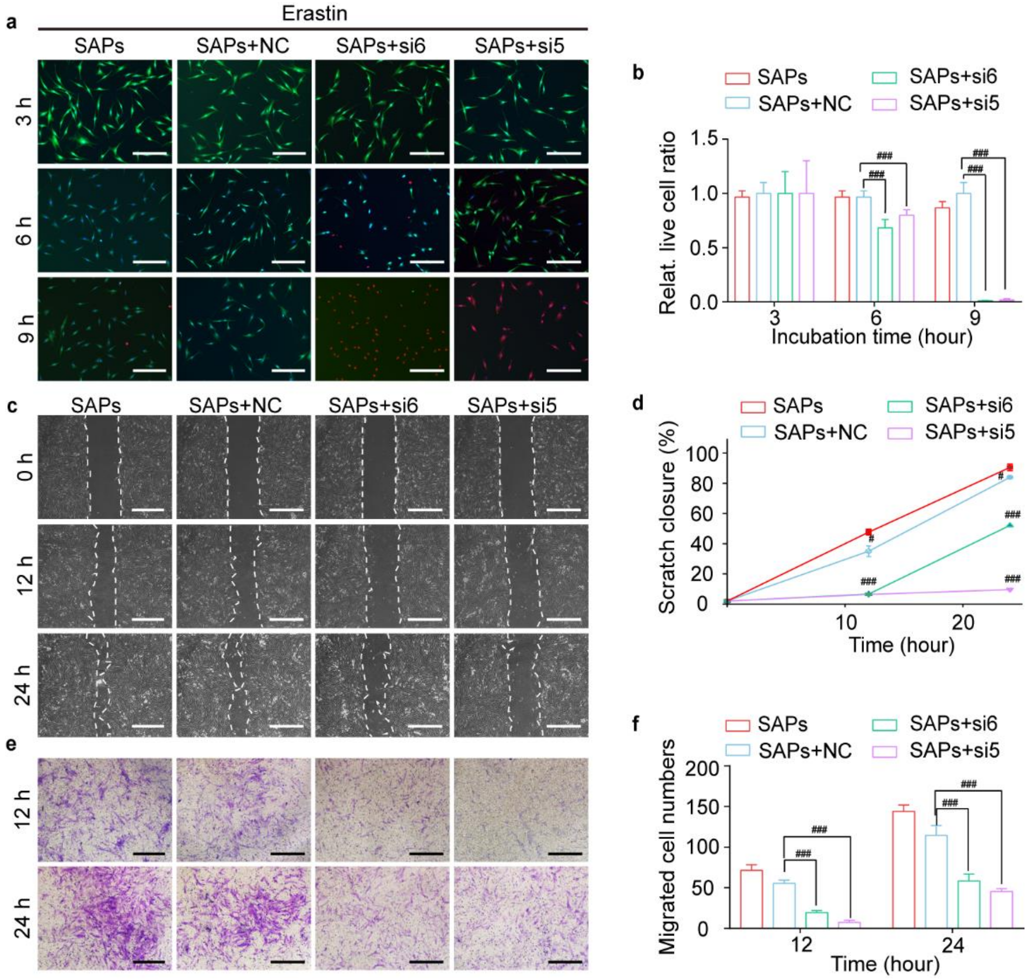

Figure S11. The influence of CHMP6 and CHMP5 on cellular functions of HG-HDFs treated with SAPs. a) and b) Representative fluorescent images (a) and quantitative

analysis (b) showing the cell viability of HG-HDFs treated with SAPs, SAPs+NC, SAPs+si6, or SAPs+si5 with the existence of erastin (10 μM, the activator of ferroptosis) (n = 3 per group). Dead cells, live cells and cell nuclei were labeled with red, green and blue fluorescence, respectively. c) and d) Representative migrating images (c) and quantitative statistics (d) showing scratch closure ratio of HG-HDFs co-cultured with SAPs, SAPs+NC, SAPs+si6, or SAPs+si5 demonstrated by scratch assay. White dotted lines indicated the boundary of migration (n = 3 per group). e) and f) Representative images (e) and matching statistical analysis (f) showing the number of migrating HG-HDFs treated with SAPs after sliencing the expression of CHMP6 and CHMP5 reflected by transwell assay. The migrated HG-HDFs were dyed with crystal violet (n = 3 per group). Data represent mean ± SD. Statistical significance was calculated by one-way ANOVA with Tukey's significant difference multiple comparisons for (b), (d) and (f). Significant differences between SAPs+NC group with other groups are indicated as $^{\#}p < 0.05$, $^{\#\#\#}p < 0.001$. Scale bar, 100 μm for (a) and (e), 200 μm for (c).

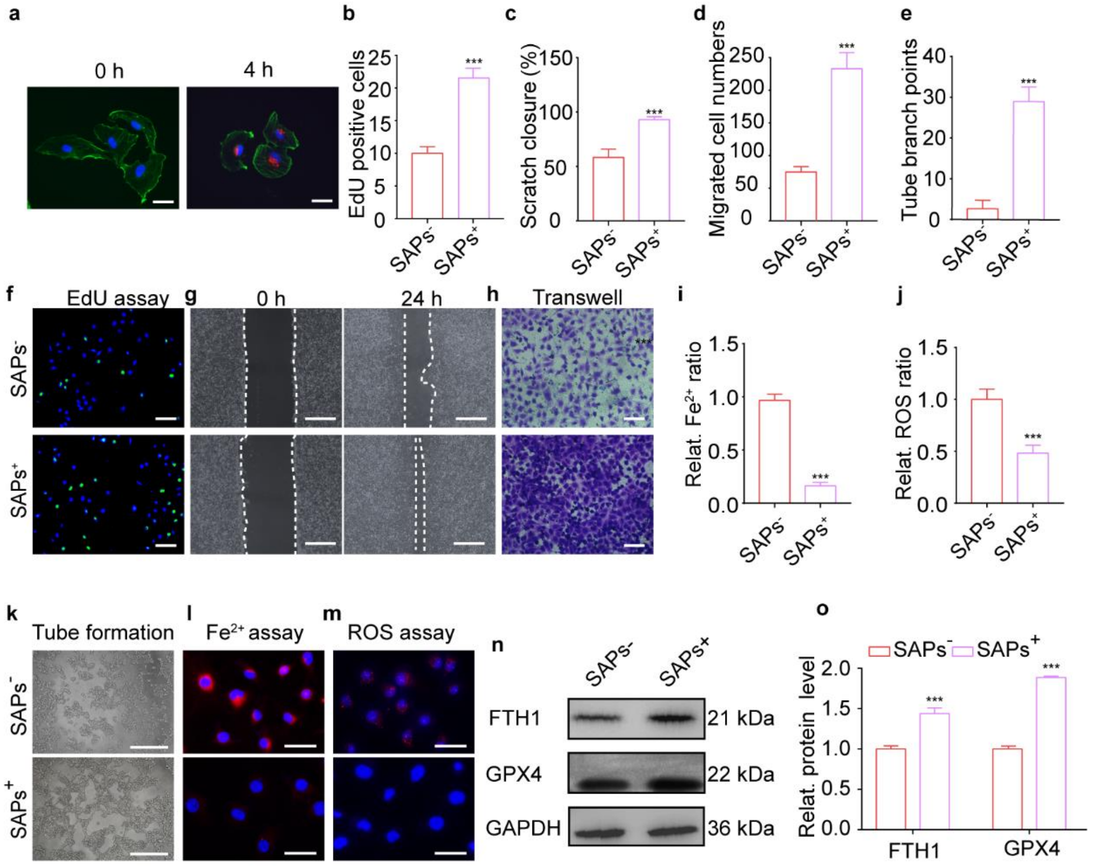

Figure S12. SAPs restored cellular behaviors of HG-HUVECs by inhibiting ferroptosis. a) Fluorescent images showing internalization of SAPs by HG-HUVECs after co-culturing for 4 h (n = 3 per group). Cell nuclei, filamentous actin and SAPs were labeled with hoechst-33342 (blue fluorescence), F-actin (green fluorescence) and Dil (red fluorescence), respectively. b) and f) EdU immunofluorescence images (f) and corresponding statistical analysis (b) indicating the proliferation behavior of HG-HUVECs treated by SAPs (n = 3 per group). Blue and green fluorescence represent hoechst-33342 staining and EdU staining, respectively. c) and g) Representative migrating images (g) and quantitative statistics (c) showing scratch closure ratio of HG-HUVECs co-cultured with SAPs demonstrated by scratch assay. White dotted lines

indicated the boundary of migration (n = 3 per group). d) and h) Representative images (h) and matching statistical analysis (d) showing the number of migrating HG-HUVECs co-cultured with SAPs reflected by transwell assay. The migrated HG-HUVECs on the back of upper chamber of the transwell system were dyed with crystal violet (n = 3 per group). e) and k) Representative images (k) and statistical analysis (e) showing the tube formation assay of HG-HUVECs treated by SAPs (n = 3 per group). i) and l) Representative fluorescent images (l) and quantitative analysis (i) of intracellular Fe^{2+} level in HG-HUVECs exposed to 3 mM SAPs after 24 h. Intracellular Fe^{2+} and cell nuclei were dyed with red and blue fluorescence, respectively (n = 3 per group). j) and m) Representative fluorescent images (j) and quantitative analysis (m) showing intracellular ROS level of HG-HUVECs after incubating with SAPs for 24 h (n = 3 per group). Intracellular ROS and cell nuclei were dyed with red and blue fluorescence, respectively. n) and o) Representative Western blotting images (n) and quantitative band intensities (o) detecting the protein expression level of FTH1 and GPX4 in HG-HUVECs stimulated with SAPs after 24 h (n = 3 per group, all proteins levels are normalized to loading control, GAPDH). Data represent mean ± SD. Statistical significance was calculated by students t test with Welchs correction for (b), (c), (d), (e), (i), (j) and (o). Significant differences between $SAPs^-$ group with other groups are indicated as $***p < 0.001$. Scale bar, 25 μm for (a), 50 μm for (k), (l) and (m), 100 μm for (f), (g), (h).

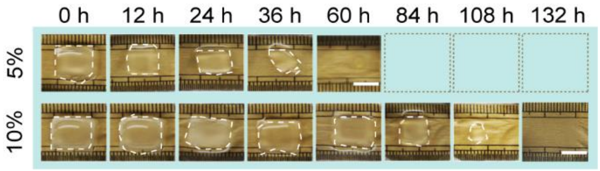

Figure S13. Representative images showing the degradation of 5%-Gel and 10%-Gel *in vitro* (n = 3 per group). Scale bar = 1 cm.

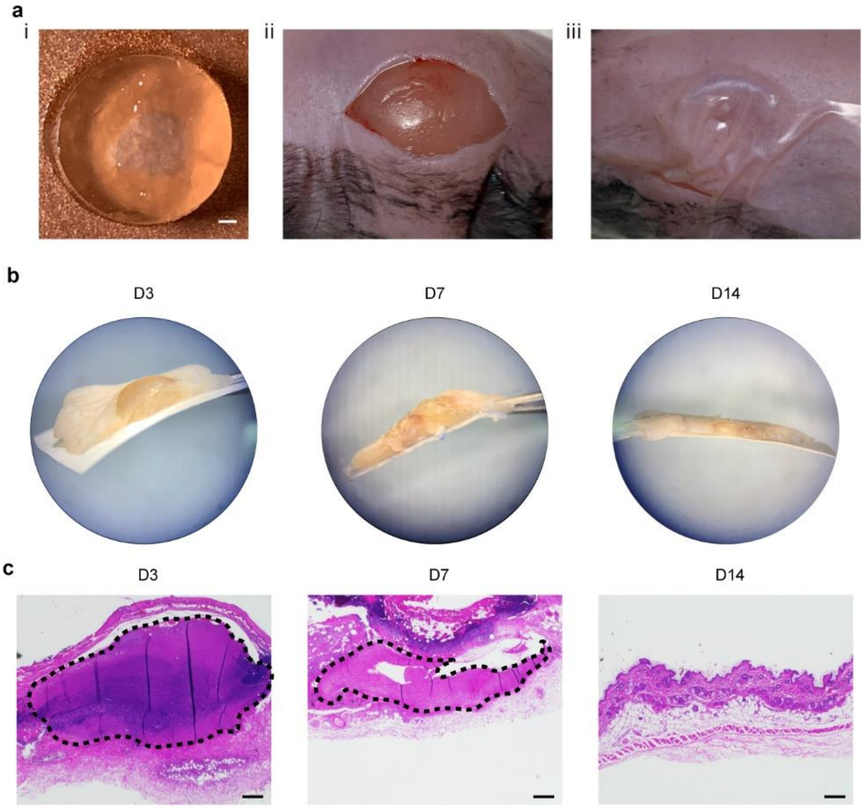

Figure S14. *In vivo* degradation study of the Gel-SAPs. a) The construction of subcutaneously implanted Gel-SAPs hydrogel model mouse. b and c) The gross appearance (b) and H&E staining (c) of Gel-SAPs at D3, D7 and D14 (black dotted line show the dividing line between materials and tissue, n = 6 per group). Scale bar, 500 μm for (ai), 100 μm for (c).

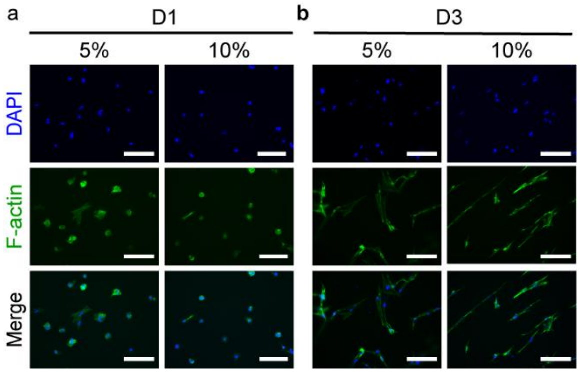

Figure S15. The fluorescent images showing elongation of HG-HDFs on 5%-Gel and 10%-Gel at D1 (a) and D3 (b) (*n* = 3 per group). The F-actin staining (green fluorescence) showed the elongation of HG-HDFs and the cell nuclei were dyed blue fluorescence. Scale bar, 100 μm.

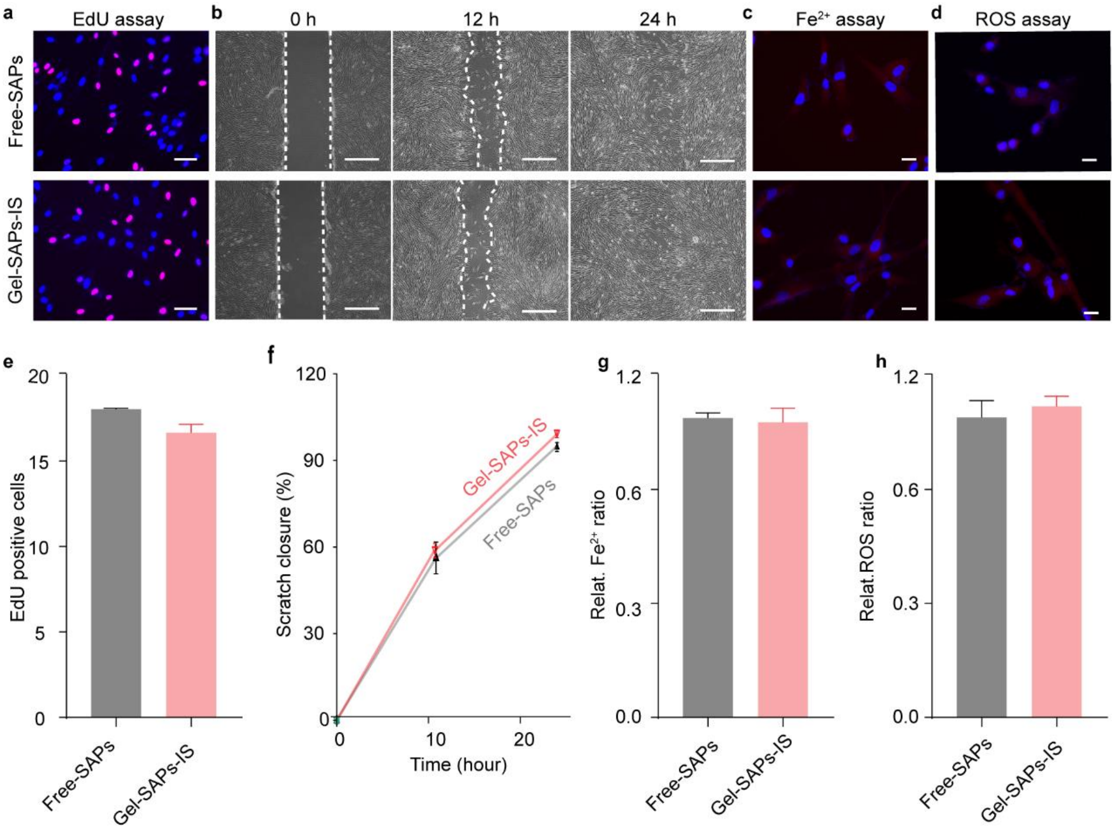

Figure S16. Photo-crosslinking and residue initiator of GelMA cannot reduce the activity of SAPs. a) and e) EdU immunofluorescence images (a) and corresponding statistical analysis (e) indicating the proliferation behavior of HG-HDFs treated by free SAPs or Gel-SAPs-IS (n = 3 per group). Blue and green fluorescence represent hoechst-33342 staining and EdU staining, respectively. b) and f) Representative migrating images (b) and quantitative statistics (f) showing scratch closure ratio of HG-HDFs co-cultured with free SAPs or Gel-SAPs-IS demonstrated by scratch assay (n = 3 per group). White dotted lines indicated the boundary of migration. c) and g) Representative fluorescent images (c) and quantitative analysis (g) of intracellular Fe^{2+} accumulation in HG-HDFs exposed to free SAPs or Gel-SAPs-IS after 24 h. Intracellular Fe^{2+} and cell nuclei were dyed with red and blue fluorescence, respectively (n = 3 per group). d) and h) Representative fluorescent images (d) and quantitative analysis (h) showing

intracellular ROS level of HG-HDFs after incubating with free SAPs or Gel-SAPs-IS for 24 h (n = 3 per group). Intracellular ROS and cell nuclei were dyed with red and blue fluorescence, respectively. Data represent mean ± SD. Statistical significance was calculated by students t test with Welchs correction for (e-h). Scale bar, 50 μm for (a), (c) and (d), 100 μm for (b).

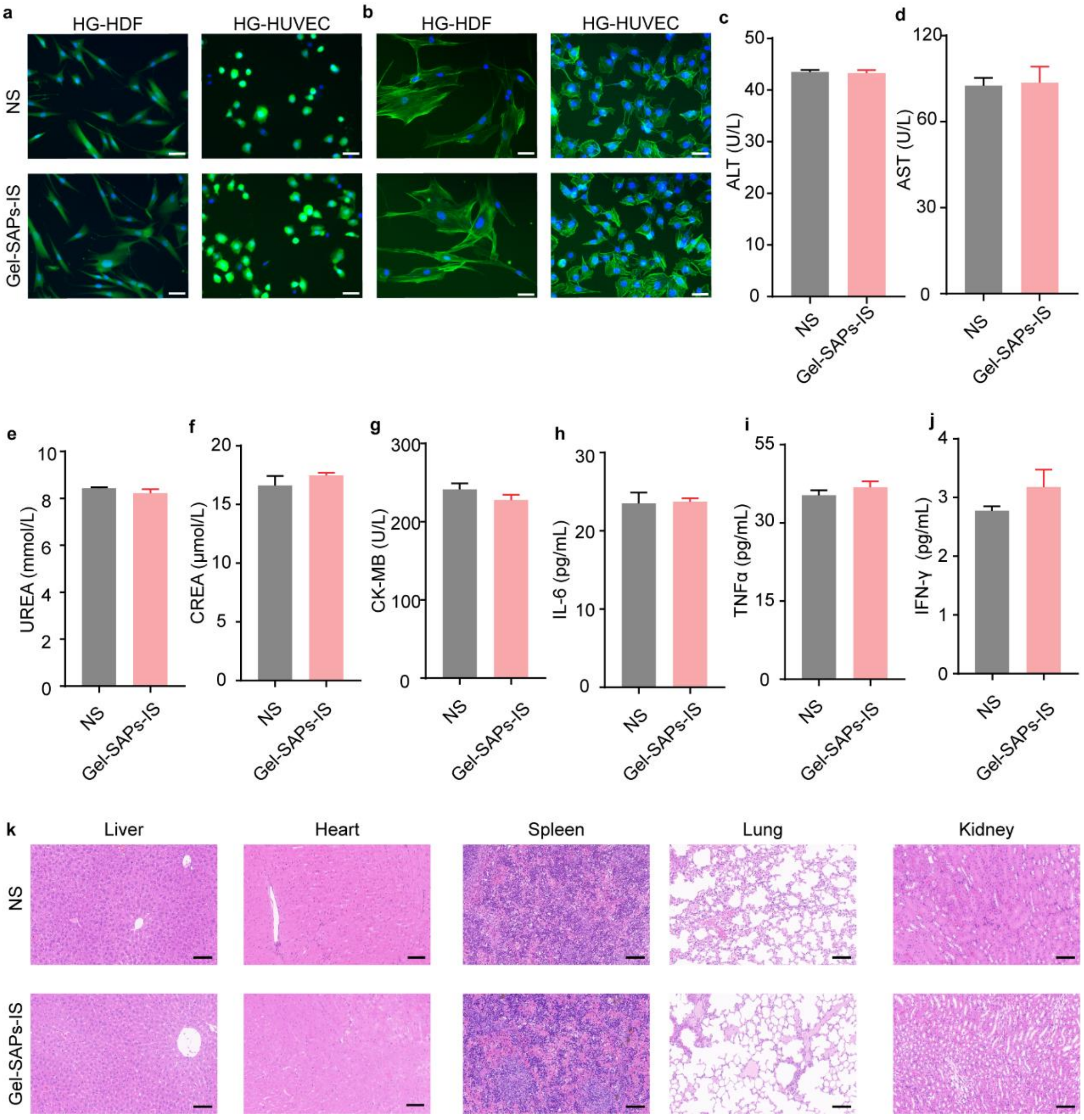

Figure S17. The biocompatibility and immunogenicity of Gel-SAPs-IS. a) Representative fluorescent images showing the live cell of HG-HDFs and HUVECs treated with PBS and the Gel-SAPs-IS (n = 3 per group). b) Representative fluorescent images showing the cytoskeleton of HG-HDFs and HUVECs treated with PBS and the Gel-SAPs-IS (n = 3 per group). c) to j) The blood biochemistry indicating of ALT, AST, UREA, CREA, CK-MB, IL-6, TNF-α and IFN-γ in Gel-SAPs-IS group were in the normal reference range (n = 6 per group). k) The histological sections of the vital internal organs. There were no obvious abnormalities in Gel-SAPs-IS and control

groups (*n* = 6 per group). Data represent mean ± SD. Statistical significance was calculated by students t test with Welchs correction for (c-j). Scale bar, 100 μm for (a) and (b), 200 μm for (k).

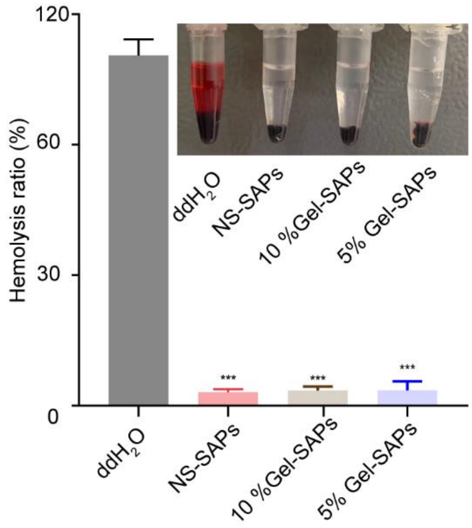

Figure S18. The Hemolysis test of NS-SAPs, 10% Gel-SAPs and 5% Gel-SAPs (n = 3 per group). Data represent mean ± SD. Statistical significance was calculated by one-way ANOVA with Tukey's significant difference multiple comparisons. Significant differences between ddH_2O with other groups are indicated as ***$p < 0.001$.

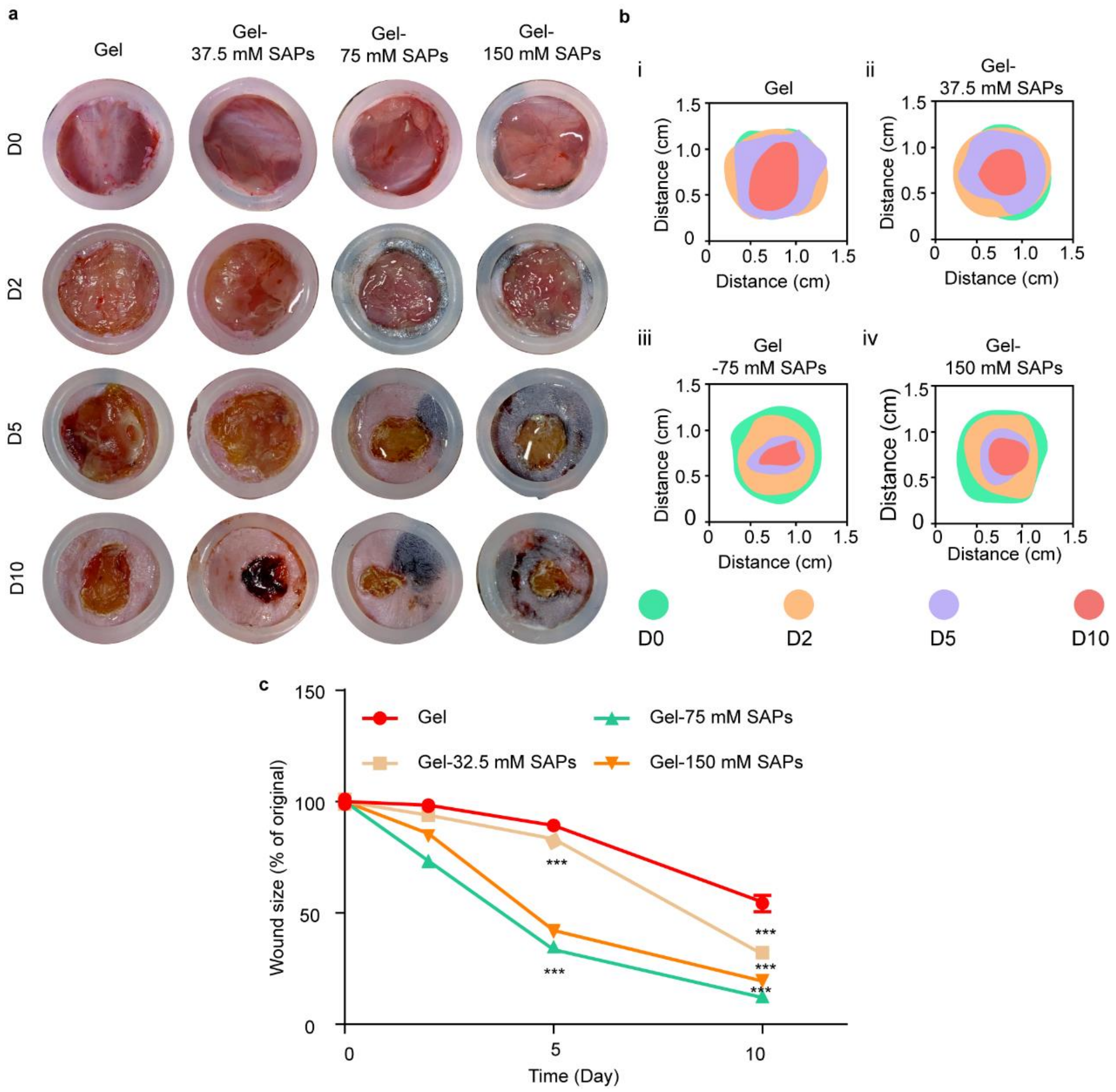

Figure S19. a)-c) Representative images (a), closure traces (b) and quantitative analysis (c) of wound closure at D0, D2, D5 and D10 treated with 0 mM, 37.5 mM, 75 mM and 150 mM SAPs (*n* = 6 per group). Data represent mean ± SD. Statistical significance was calculated by one-way ANOVA with Tukey's significant difference multiple comparisons. Significant differences between Gel group with other groups are indicated as ***$p < 0.001$.

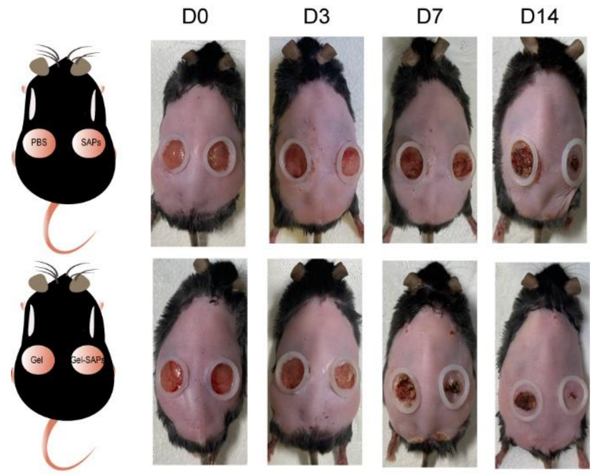

Figure S20. The whole mice figure of diabetic wound model in DB/db mice (*n* = 6 per group).

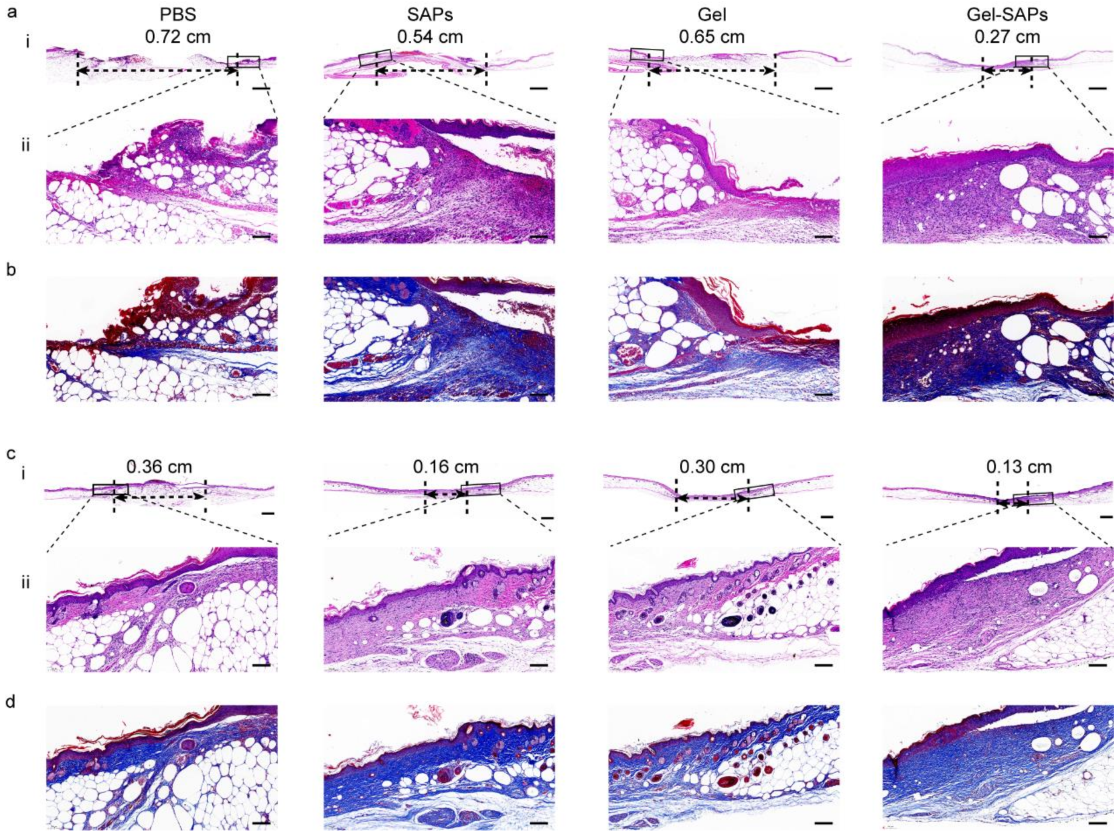

Figure S21. H&E staining (ai-ii, ci-ii) and masson's trichrome staining (b, d) of wounds treated with PBS, SAPs, Gel and Gel-SAPs at D7 and D14 (*n* = 6 per group). Scale bar, 1000 μm (ai), 100 μm for (ci), 100 μm for (aii, b, cii, d).

Table S1: Sequences of *siRNA* used for transfection.

Gene	Target sequence
CHMP6-1	CGCGCAATCACTCAGGAACAAA
CHMP6-2	GCAATCACTCAGGAACAAATA
CHMP5-1	AAGCGAAACCCAAGGCTCC
CHMP5-2	AAGGACACCAAGACCACGGTT

Table S2: The sequences of primers used.

Gene	Primer sequence
CHMP6-F	AAGGCCATCCTGCAACTGAAG
CHMP6-R	GCTGCTCCTGGTATCGCTT
CHMP5-F	GACACCAAGACCACGGTTGAT
CHMP5-R	GGGTGCATAACTGCGACTC
DDIT3-F	GGAAACAGAGTGGTCATTCCC
DDIT3-R	CTGCTTGAGCCGTTCATTCTC
ATF4-F	CCCTTCACCTTCTTACAACCTC
ATF4-R	TGCCCAGCTCTAAACTAAAGGA
GAPDH-F	GGAGCGAGATCCCTCCAAAAT
GAPDH-R	GGCTGTTGTCATACTTCTCATGG
